# Oxygen‐Regulated GaN‐Based Sensors Fabricated by MOCVD for Switchable Gas Detection: Exhaled Gas Smart Platform for Non‐Invasive Disease Detection

**DOI:** 10.1002/advs.202515282

**Published:** 2025-11-19

**Authors:** Yuxuan Wang, Dan Han, Qi Duan, Zhekai Zhang, Zhengyang Jia, Juxu Guang, Guojing Wang, Weidong Wang, Xiuli He, Shengbo Sang

**Affiliations:** ^1^ Shanxi Key Laboratory of Micro Nano Sensors & Artificial Intelligence Perception College of Integrated Circuits Taiyuan University of Technology Taiyuan Shanxi 030024 China; ^2^ State Key Laboratory of Transducer Technology Aerospace Information Research Institute Chinese Academy of Sciences Beijing 100190 China; ^3^ Medical Innovation Research Division Chinese PLA General Hospital Beijing 100853 China; ^4^ Key Laboratory of Biomedical Engineering and Translational Medicine Ministry of Industry and Information Technology Chinese PLA General Hospital Beijing 100853 China

**Keywords:** chemical adsorption of oxygen, GaN, multi‐gas detection, non‐invasive disease detection, signal mapping

## Abstract

Miniaturized, easily integrated thin‐film gas sensors with high consistency, stability, and suitability for mass production are crucial for non‐invasive breath analysis. A series of GaN‐based thin‐film sensors with high stability and excellent sensing capabilities prepared by Metal‐organic Chemical Vapor Deposition, in which the n‐GaN sensor exhibited an ultra‐low detection limit of 100 ppt for NO_2_, and 2 ppb for NO, the InGaN sensors with different In composition manifest good response to reducing gases of NH_3_ and TMA. The shift trend of selectivity for the sensors from oxidizing gases to reducing gase. can be attributed to the mutative relative content of the lattice oxygen/adsorbed oxygen ratio by adjusting the In composition, which is based on the in situ characterization results. The sensors exhibit excellent hydrophobicity, significantly enhancing their reliability in exhale gas sensing under high‐humidity conditions. Response mapping model through mixed‐gas training improved cross‐sensor compatibility and enhanced model generalization. Herein an integrated multimodal sensor system combining a sensor array with a temperature and humidity sensor is proposed and developed alongside a dual‐channel exhaled breath collection system. Utilizing a Time Sequence Analysis model, this integrated system enables non‐invasive diagnosis of lung cancer patients.

## Graphical abstract

This work presents highly consistent GaN‐based thin‐film gas sensors prepared by MOCVD for non‐invasive breath analysis. The sensors exhibit tunable selectivity from oxidizing to reducing gases by incorporating In composition, ultra‐low detection limits and humidity resistance. An integrated multimodal system combining a sensor array with a Time Sequence Analysis model enables accurate lung cancer diagnosis from exhaled breath.

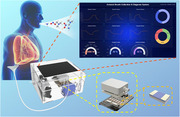

## 1 Introduction

Gas sensing and recognition technology is critical for social development and technological progress, directly contributing to environmental conservation,^[^ ^1^ ^]^ energy efficiency,^[^ ^2^ ^]^ and public health safety.^[^ ^3^ ^]^ Within the public health domain, exhaled breath analysis drives a transformative shift toward precise, non‐invasive diagnostics, particularly through breathomics‐enabled biomarker detection. Exhaled breath biomarkers demonstrate significant potential for early cancer detection through metabolic fingerprinting techniques, capturing underlying biochemical alterations associated with lung cancer (LC),^[^ ^4^ ^]^ asthma,^[^ ^5^ ^]^ pneumonia,^[^ ^6^ ^]^ and diabetes^[^ ^7^ ^]^ through analytical platforms. Sensor technology constitutes the foundation for analytical platforms, while the recent advances in materials science have expanded the options for sensor sensing elements, and the integration of artificial intelligence with sensor arrays has significantly enhanced diagnostic accuracy by improving analytical precision, reliability, and repeatability.^[^ ^8^, ^9^ ^]^

Emerging materials like Ti_3_C_2_T_X_@Cu_3_(HHTP)_2_^[^ ^3^ ^]^ and ZnO/ZnSnO_3_^[^ ^10^ ^]^ are increasingly employed in gas sensor cores. Morever, the Muhammad Hilal et al.^[^ ^11^ ^]^ through defunctionalize and functionalize Mxenes with elemental forms of functional groups (–I and –Br), Mxene sensors for NO_2_ detection with an excellent detection limit were fabricated. Fan Yang et al.^[^ ^12^ ^]^ synthesized Pt‐nanoparticle‐decorated highly permeable Bi_2_O_3_ microspheres using a facile template method, achieving acetic acid detection at a relatively low operating temperature. However, most sensors exhibit limited efficacy due to material instability, challenges in precise structural/defect control, and thickness uniformity issues. Semiconductor metal oxides avoid complex synthesis but suffer from poor consistency, high operating temperatures, and power consumption. Collectively, these limitations impede ultra‐trace detection, miniaturization, and multi‐gas analysis, restricting practical deployment. In contrast, GaN demonstrates superior sensing performance due to its exceptional chemical stability and unique electrical properties. The GaN is typically grown through metalorganic chemical vapor deposition (MOCVD). Performance enhancement strategies for GaN include structural design and surface modifications. Shanmugasundaram et al.^[^ ^13^ ^]^ developed InGaN/GaN multi‐quantum well nanowires achieving 5 ppb NO_2_ detection limit. while Baek et al.^[^ ^14^ ^]^ created AlGaN/GaN HEMT synapses with Pd/graphene gates for NO_2_ sensing. Despite promising performance in residual gas detection, GaN‐based sensors face fundamental constraints of narrow selectivity and low sensitivity in mixed‐gas environments.

Integration of gas sensors with AI for multi‐gas monitoring constitutes an emerging paradigm in smart sensing. While traditional algorithms (SVM,^[^ ^15^, ^16^ ^]^ KNN,^[^ ^17^, ^18^ ^]^ and LDA^[^ ^19^, ^20^ ^]^) effectively discriminate single gases using response features such as magnitude, response/recovery times. Traditional machine learning algorithms face challenges in detecting mixed gas types and concentrations in complex multi‐gas environments due to intermolecular interactions among the gases when relying solely on individual response features. Deep learning algorithms overcome this limitation through enhanced adaptability, enabling precise mixed‐gas analysis as demonstrated by Juan Li et al.^[^ ^21^ ^]^ for simultaneous quantification of NO_2_ NH_3_, CH_4_ and CO_2_. Yang Jiang et al.^[^ ^22^ ^]^ achieved high‐precision hydrogen concentration recognition in complex gas environments by integrating a hydrogen sensor with reservoir computing. Recent advances confirm AI‐optimized sensor arrays can identify cross‐sensitive multi‐component mixtures. However, sensor‐specific response characteristics impede cross‐sensor data interoperability, creating closed ecosystems incompatible with LLM and CV data paradigms.

**Figure 1:**
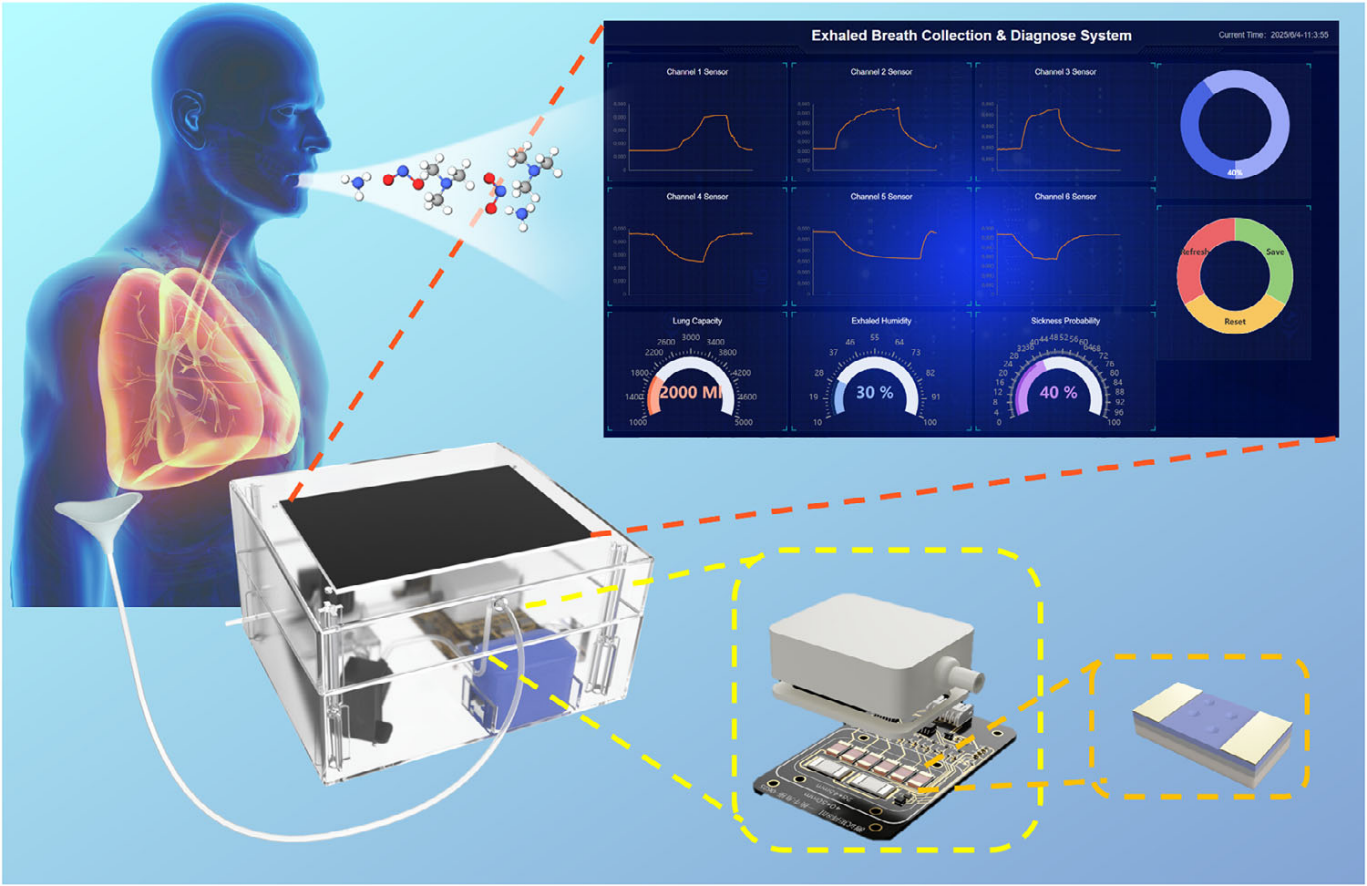
Exhaled breath collection and diagnose system.

In this work, a controllable fabrication method through MOCVD for building a series of thin‐film gas sensors for oxidizing/reducing gas detection, including a n‐type GaN film sensor with a thickness of 20 nm (n‐GaN‐20), the InGaN film sensor with a thickness of 20 nm (InGaN‐20), the InGaN film sensor with a thickness of 15 nm (InGaN‐15) and the InGaN film sensor with high In component content (InGaN‐High). The n‐GaN‐20 demonstrates excellent performance on NO_2_ detection with the ultra‐low detection limit of 100 ppt and 2 ppb for NO detection. Meanwhile, the n‐GaN‐20 demonstrated good repeatability, long‐term stability and hydrophobic ability. Furthermore, the InGaN gas sensors were fabricated by incorporating In components to investigate the effects of the In component on sensing performance. With the increase of In component content, InGaN sensor with high In component exhibits low detection capability towards oxidizing gases while demonstrating responsive behavior to reducing gases. To further analysis the In component regulation mechanism, in situ characterization was employed for mechanistic analysis of gas‐sensing behavior. In situ analysis revealed that the In component modulates the relative ratio of lattice oxygen and absorbed oxygen, modulating the redox selectivity of the InGaN sensors, establishing a gas‐selective sensitivity enhancement mechanism. To achieve the compatibility of the detection model for different sensors, a response signal mapping model was trained with mixed gas response dataset to achieve the mapping of response signals from different sensors, which can effectively improve the generalization ability of the post recognition model. Finally, a dual‐channel exhaled breath collection system integrated with the fabricated sensor array was constructed, capable of collecting breath samples from patients and healthy individuals. A Time Sequence analysis model was trained for non‐invasive disease detection through breath analysis presented as **Figure** **1**.

## 2 Results

### 2.1 Structure Characterization

To analyze the surface morphology of the prepared GaN‐based film, SEM characterization was conducted. Hexagonal pits were observed on the surfaces of InGaN‐20 and InGaN‐15, whereas the n‐GaN‐20 surface appeared flat (Figure S2, Supporting Information). In order to further analyze the cross sectional of samples, we cut the samples through the focused ion beam (FIB) for STEM characterization. STEM analysis of cross‐sectional views (Figure S3, Supporting Information) reveals distinct stratification in the n‐GaN‐20, InGaN‐20, and InGaN‐15 samples, consistent with expectations.

**Figure 2:**
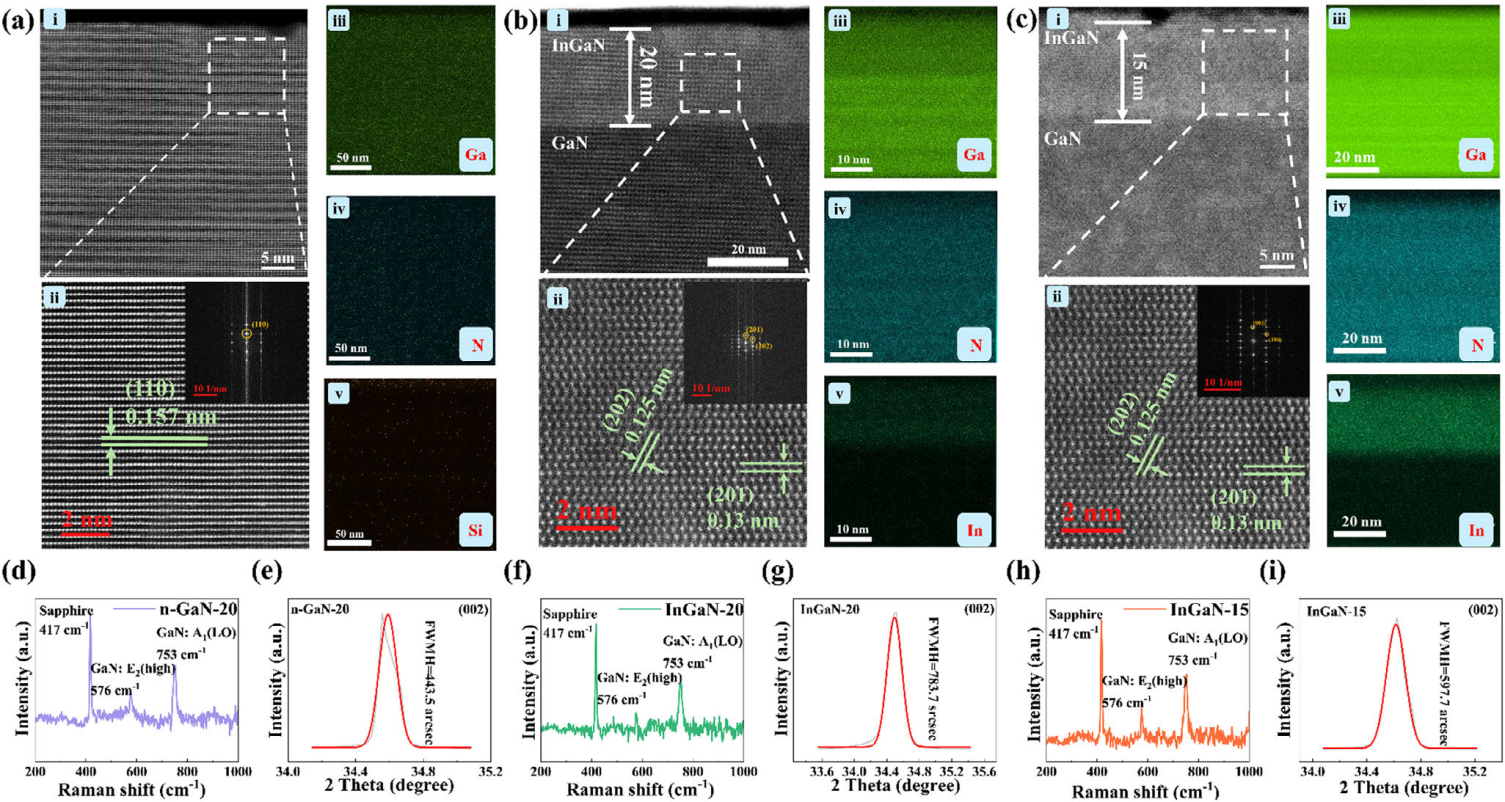
Films crystal quality analysis. STEM and HRTEM images results of the a) n‐GaN‐20, b) InGaN‐20 and c) InGaN‐15. The Raman results of the d) n‐GaN‐20, f) InGaN‐20, and h)InGaN‐15. XRD result of the e) n‐GaN‐20, g) InGaN‐20 and i) InGaN‐15.

**Figure** **2**a–c shows the STEM and HRTEM of each sample. The thickness of the InGaN films for the InGaN‐15 and InGaN‐20 is ≈ 15 and 20 nm, respectively, which conforms to the anticipated values. Furthermore, the cross‐sectional EDS analysis confirms that the element composition of InGaN films for the InGaN‐20 (Figure 2b iii‐v) and InGaN‐15 (Figure 2c iii‐v) consists of Ga, N and In, the film of the n‐GaN‐20 (Figure 2a iii‐iv) consists of Ga and N. Additionally, the HRTEM images show that the lattice fringes exhibit a highly ordered, continuous, and regular arrangement, which can be concluded that the samples produced in this work are single‐crystalline. The (110) planes for hexagonal GaN (JCPDS 00‐002‐1078) where interplanar spacing is 0.157 nm was identified in the GaN layer of the n‐GaN‐20 (Figure 2a ii). For the samples of the InGaN‐20 (Figure 2b ii), the interplanar spacing of (201) and (202) planes of the InGaN layer are 0.13 and 0.125 nm, respectively. The (002) and (100) planes of hexagonal GaN were identified in the InGaN‐15 (Figure 2c ii). To further reveal the surface structure for each sample, the Raman spectra of the n‐GaN‐20 (Figure 2d), InGaN‐20 (Figure 2f) and InGaN‐15 (Figure 2f) are recorded. The result of Raman test shown sapphire (Al_2_O_3_) substrate peak at 417 cm^−1^ and GaN phonon modes: E_2_^(high)^ at 576 cm^−1^, A_1_^(LO)^ at 753 cm^−1^.^[^ ^23^ ^]^ Furthermore, the test result shows that the E_2_^(high)^ mode exhibits a compressive stress‐induced upshift relative to unstrained GaN,^[^ ^24^, ^25^ ^]^ indicating the presence of surface defects and lattice mismatch in each samples.

The XRD was utilized to characterize the crystal quality of the composite films and compare the dislocation density in the GaN‐based films, as analyzed through the FWHM of the (002) ω‐rocking curve. All samples exhibited high crystallinity, with the n‐GaN‐20 (Figure 2d) shows narrower FWHM than that of the InGaN‐20 (Figure 2f) and InGaN‐15 (Figure 2h), indicating reduced screw dislocation density and consequently smoother surfaces. The result of XRD reveal that the FWHM of (002) for the InGaN‐20 is wider than that of the InGaN‐15, consistent with higher surface defect density of SEM result. Consistent XRD and SEM results confirm that In incorporation degrades the crystalline quality of GaN‐based film. The existence of (002) diffraction peak intensity confirms wurtzite structure and polar nature. The (102) plane measurement was precluded due to ultra‐thin cap layer thickness. Additionally, X‐ray photoelectron spectroscopy (XPS) was employed to investigate the electron structure. As depicted in Figure S4 (Supporting Information), the n‐GaN‐20 exhibited abundant adsorbed oxygen content, with the incorporation of the In component, the adsorbed oxygen content decreased while the lattice oxygen content increased. The detailed analysis is described in Note S7 (Supporting Information).

### 2.2 Gas Sensing Characteristics

The performance of gas detection of sensors was measured at RT (27°C) and 30% RH. The sensing response of the sensor was calculated using the following equation:

(1) $$\mathrm{Resp}\mathrm{onse}\left(\%\right)=\frac{\left|R_{g}-R_{a}\right|}{R_{a}}\times 100\%$$

where R_a_ and R_g_ represent the initial and real‐time sensor resistances after the introduction of the target gas. The selectivity and recovery response time were tested to evaluate the gas sensing performance of the n‐GaN‐20, InGaN‐20, InGaN‐15.

**Figure 3:**
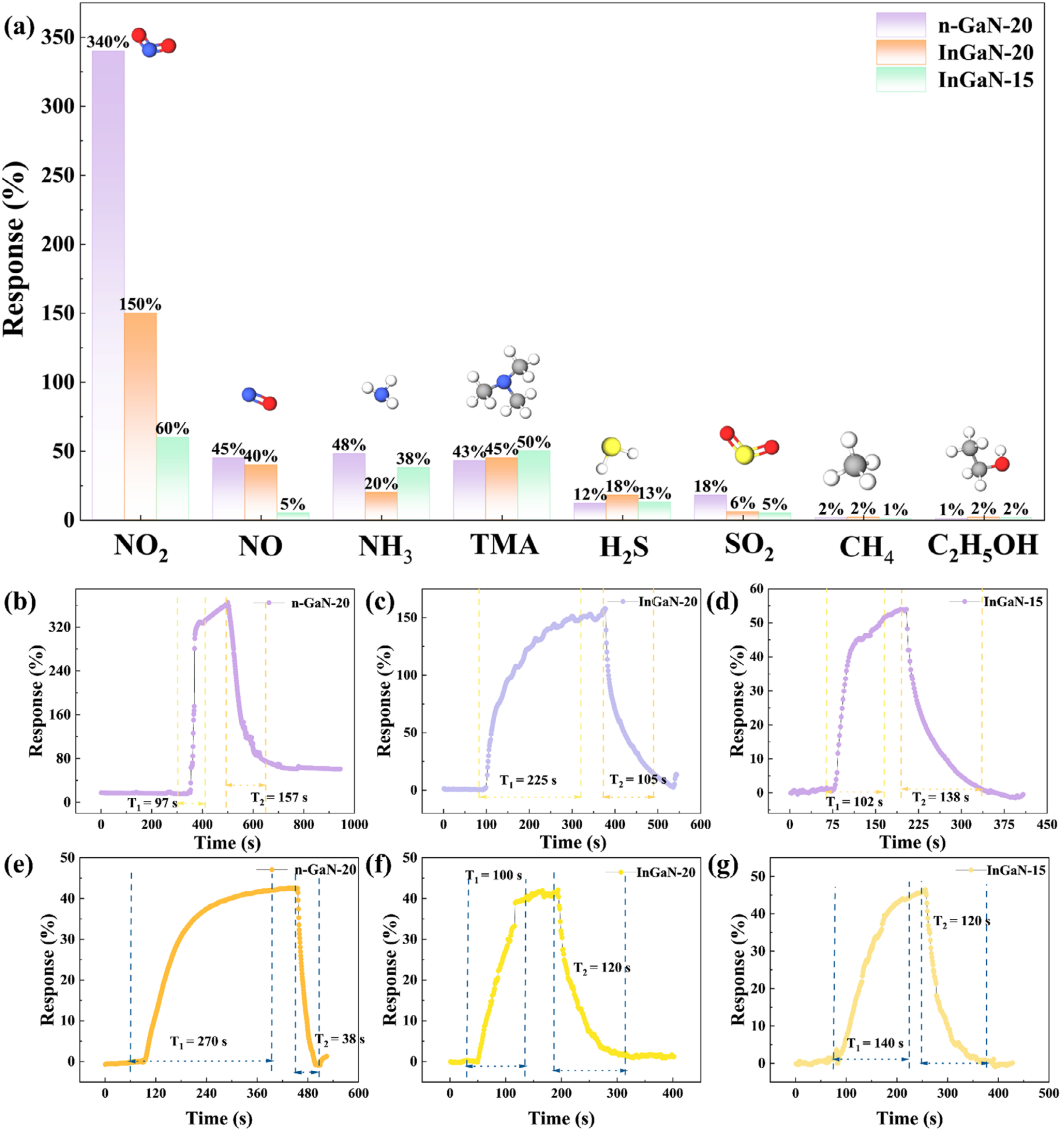
a) Selectivity of n‐GaN‐20, InGaN‐20, InGaN‐15 toward 200 ppm different gases at RT. Response and recovery time of oxidizing gas for the b) n‐GaN‐20, c) InGaN‐20 and d) InGaN‐15. Response and recovery time reducing gas for the e) n‐GaN‐20, f) InGaN‐20 and g) InGaN‐15.

According to literature,^[^ ^26^, ^27^ ^]^ selectivity is defined as (i) sensitivity to specific species or groups while maintaining insensitivity to interferents, or(ii) the generation of distinctive patterns that discriminate between analyte types. In this work, eight types of gases (NO_2_, NO, NH_3_, TMA, H_2_S, SO_2_, CH_4_, and ethanol) were tested at a concentration of 200 ppm, with the results presented in **Figure** **3**a. Compared to nonpolar gas, the n‐GaN‐20, InGaN‐20, and InGaN‐15 exhibit enhanced selectivity toward polar gas molecules through dipole‐dipole interactions facilitated by abundant polar Ga‐N bonds. Furthermore, nitrogen‐containing gas molecules exhibit enhanced responses relative to other gases, which due to the nitrogen atoms possess lone pair electrons that enhance coordination with GaN surface vacancies, thereby improving adsorption stability.

The response time (T_1_) and recovery time (T_2_) are another critical parameters of gas sensor, which refers to the time required for response value to rise from 10% to 90% or fall from 90% to 10%.^[^ ^28^ ^]^ For oxidizing gas of NO_2_ at 200 ppm, n‐GaN‐20 (Figure 3b) exhibited faster T_1_ and T_2_ (97 s and 157 s, respectively) than that of InGaN‐20 (Figure 3c, 225 s, 105 s) and InGaN‐15 (Figure 3d, 135 s, 168 s). This faster T_1_ for n‐GaN‐20 correlates with its smoother surface as shown on the AFM result (Figure S4, Supporting Information), which provides less restricted pathways for gas adsorption and desorption and gas contact area, enhancing adsorption efficiency. However, it was observed that the T_2_ of the n‐GaN‐20 sample to NO_2_ was slower than that of the InGaN‐20 and InGaN‐15 samples. This is attributed to the significantly higher response magnitude of n‐GaN‐20 to NO_2_ compared to the InGaN‐based samples, where a greater response magnitude leads to an extended desorption time. Moreover, the T_1_ and T_2_ of the sensors exposed to reducing gas of TMA were indicated on Figure 5e–g. Compared to the T_1_ and T_2_ for oxidizing gas, the T_1_ of the InGaN‐20 (Figure 3f) and InGaN‐15 (Figure 3g) is faster than that of the n‐GaN‐20 (Figure 3e). The shorten of T_1_ performance arises from the elevated carrier density and enhanced material conductivity induced by In incorporation.^[^ ^29^, ^30^ ^]^ Furthermore, the test results indicate that n‐GaN‐20 exhibits a faster T_2_, which is attributed to its higher carrier mobility (as shown in the Figure S6, Supporting Information) and smoother surface. The three prepared sensors exhibited similar responses to TMA. However, the smoother surface of the n‐GaN‐20 sample facilitated the desorption process of TMA. Furthermore, the increased carrier mobility facilitates rapid diffusion of carriers released after desorption, enabling swift re‐equilibration of charge distribution and thereby shortening the recovery period.

**Figure 4:**
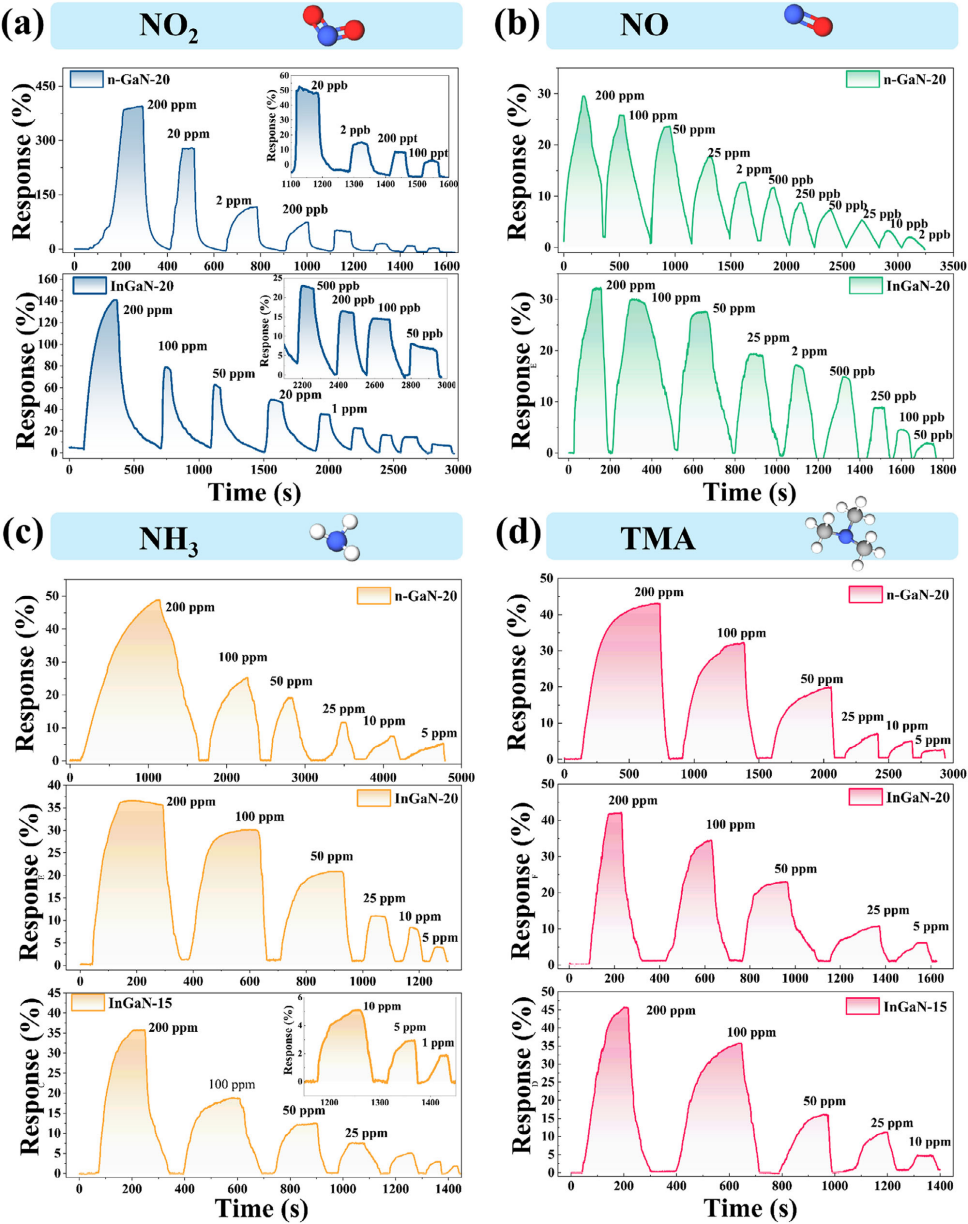
Response curves of the n‐GaN‐20 and InGaN‐20 for oxidizing gas: a) NO_2_, b) NO. Response curves of the n‐GaN‐20, InGaN‐20, and InGaN‐15 for reducing gas: c) NH_3_, (d) TMA.

**Figure** **4** and S9 (Supporting Information) provide dynamic response curves and resistance curves of different gases for the InGaN‐15, InGaN‐20, and n‐GaN‐20. In Figure S9 (Supporting Information), compared with the n‐GaN‐20, the InGaN‐20 and InGaN‐15 have lower baseline resistance, which is due to the reduced bandgap induced by the incorporation of the In component, thereby enhancing electrical conductivity. As the concentration of each gas decreases, the response of all sensors falls correspondingly, as illustrated in Figure 4, revealing that a positive correlation between sensor response and gas concentration. In Figure 4a, the n‐GaN‐20 and InGaN‐20 attain a maximum response with a response of 340% and 150% at 200 ppm NO_2_, respectively. The n‐GaN‐20 achieves an ultra‐low detection limit (LOD) of 100 ppt with 10% response value, while the InGaN‐20 exhibits LOD of 50 ppb with a 3% response value. The NO gas‐sensing measurements were conducted under N_2_ atmosphere using a dynamic gas‐blending system (Figure S11, Supporting Information) to avoid oxidation of NO to NO_2_. During NO testing, an increase in baseline resistance was observed for both the n‐GaN‐20 and InGaN‐20 sensors. This phenomenon is primarily attributed to the N_2_ purging process employed to cleanse gas pathways and reaction chambers, which prevents NO oxidation. In ambient air, water molecules adsorb on the surface of GaN‐based sensors and passivate surface states, thereby influencing the sensor's surface properties. During the purge process in dynamic testing, the introduced gas flow effectively suppresses further adsorption of water molecules on the GaN surface. The reduction in water adsorption weakens the corresponding electron transfer process. Morever, prolonged N_2_ purging can re‐expose the intrinsic GaN surface and reactivating the previously passivated surface states, leading to the increases in surface state density. These reactivated states exhibit strong electron‐trapping capabilities on the n‐type GaN surface, causing localized trapped electrons to become incapable of participating in conduction. This process induces substantial carrier capture and widens the depletion layer near the surface. The consequent reduction in free carrier density within the depletion layer ultimately leads to a marked increase in electrical resistance. The LOD for n‐GaN‐20 and InGaN‐20 is 2 and 50 ppb, respectively. The response of the InGaN‐15 for NO_2_ and NO reduced obviously owing to further increased In incorporation than that of the n‐GaN‐20 and InGaN‐20. It is interesting that the n‐GaN‐20, InGaN‐20, and InGaN‐15 demonstrated relatively good sensitivity toward NH_3_ (Figure 4c) and TMA (Figure 4d). Notably, the InGaN‐15 achieves the lowest NH_3_ detection limit with 1.6% at 1 ppm, the n‐GaN‐20 and InGaN‐20 achieves the lowest NH_3_ detection limit with 5% and 3% at 5 ppm. As measured under identical conditions, the TMA detection limits of the n‐GaN‐20, InGaN‐15, and InGaN‐20 are 1 ppm (1% response), 10 ppm (5% response) and 5 ppm (5% response), respectively, with n‐GaN‐20 achieving the lowest LOD. Theoretical detection limits were calculated for comparative analysis of the sensors performance in detecting reducing gases. The LOD toward NH_3_ for n‐GaN‐20, InGaN‐20 and InGaN‐15 were 880.5 ppb (Figure S12a, Supporting Information), 4.19 ppb (Figure S12c, Supporting Information) and 148.3 ppb (Figure S12e, Supporting Information), respectively. The LOD toward TMA for n‐GaN‐20, InGaN‐20 and InGaN‐15 were 143.9 ppb (Figure S12b, Supporting Information), 8.94 ppb (Figure S12d, Supporting Information) and 34.4 ppb (Figure S12f, Supporting Information), respectively. The calculation results of the LOD for NH_3_ and TMA show that the incorporation of In component is conducive to the detection of reducing gas.

**Figure 5:**
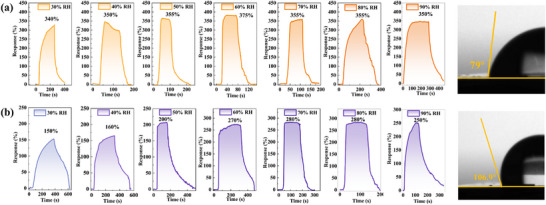
The response of NO_2_ gas sensor exposed to 200 ppm NO_2_ from 30% to 90% RH and corresponding water contact angle data of (a) n‐GaN‐20, (b)InGaN‐20.

Given the crucial role of hydrophobicity in sensors for applications like human breath analysis, the hydrophobicity and the influence of humidity on the response of the sensors were investigated. To investigate the influence of humidity on the sensors the sensors were tested with 200 ppm NO_2_ exposure at relative humidity (RH) ranging from 30% (ambient humidity) to 90% RH. In the humidity performance test of the sensor, we accurately control the humidity inside the air chamber by combining a humidity generator with a humidity sensor. When the humidity levels in the gas chamber and the baseline resistance of the sensor reach a stable state, a specific concentration of NO_2_ is introduced into the gas chamber to monitor the resulting change in the sensor's resistance value. The results demonstrate that for the n‐GaN‐20 (**Figure** **5**a) and InGaN‐20 (Figure 5b), the sensors response to 200 ppm NO_2_ increase consistently as the RH rises from 30% to 90%. To gain deeper insight into these differences, the hydrophilicity and hydrophobicity of the sensors were analyzed through water contact angle measurements. The results indicate that the InGaN‐20 and n‐GaN‐20 show good hydrophobic properties. The hydrophobic surfaces of InGaN‐20 and n‐GaN‐20 inhibit water film formation, and water molecules provide additional elections to promote proton (H^+^) hopping,^[^ ^31^ ^]^ leading to an increased response with higher humidity. Water contact angle measurements confirm the inherent hydrophobicity of our GaN‐based sensors, which effectively suppresses bulk water condensation on the sensing surface under high‐humidity conditions. This hydrophobic barrier prevents the formation of continuous water films that would otherwise impede gas diffusion and analyte adsorption. Morever, based on the proton (H^+^) hopping, trace moisture undergoes dissociation at chemisorbed NO_2_ sites, forming adsorbed HNO_3_ species. The resulting acidified interface generates mobile H+ ions that propagate along defect pathways such as nitrogen vacancies in the GaN lattice pathways. These protons electrostatically modulate the depletion layer depth, enhancing charge transfer efficiency beyond the limitations of pure NO_2_ chemisorption. This synergistic mechanism thereby amplifies sensor response in humid environments.

**Figure 6:**
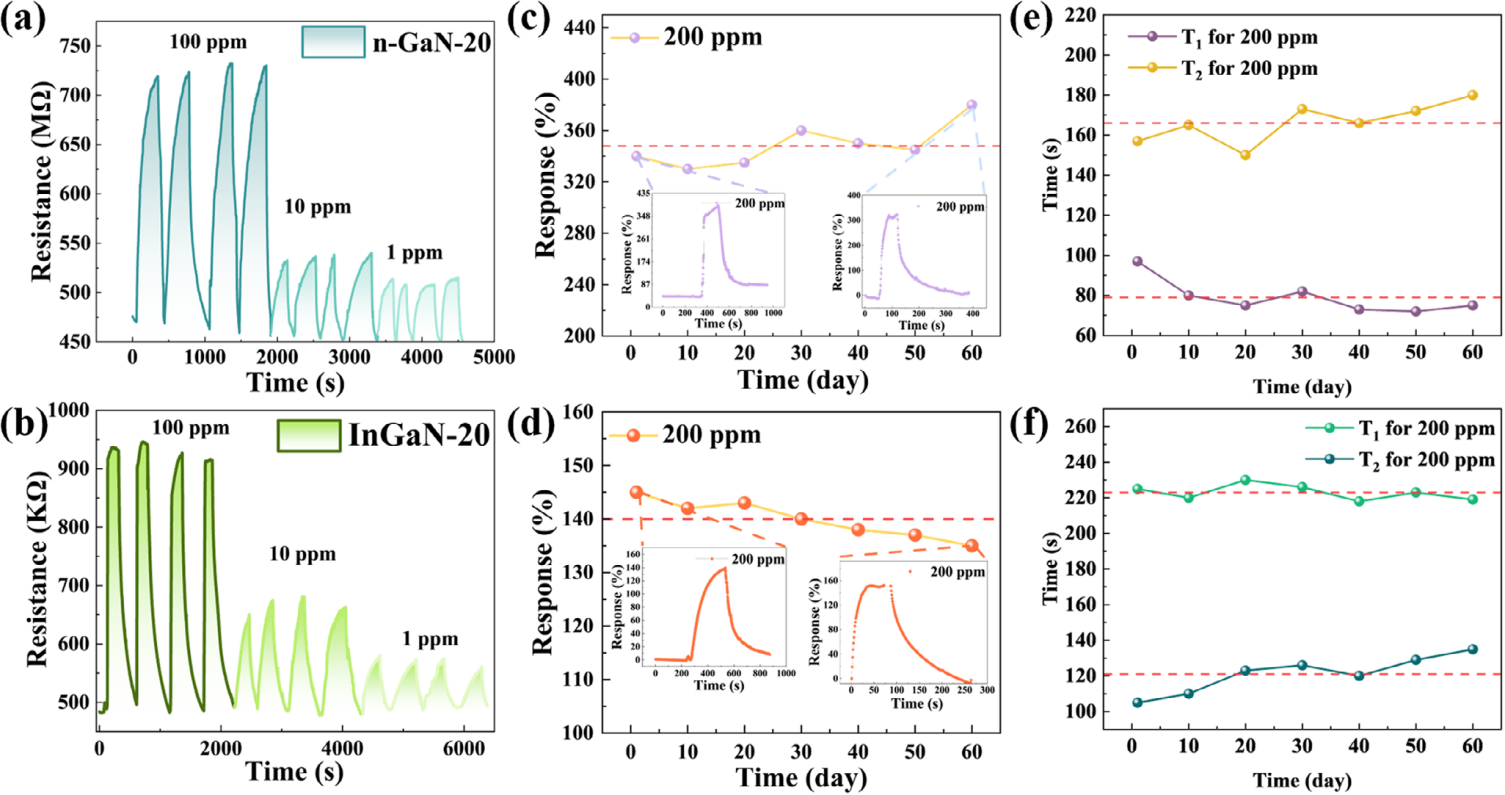
Repeatability of a) n‐GaN‐20 and b) InGaN‐20 for 100, 10, and 1 ppm NO_2_. long‐term stability for the c) n‐GaN‐20 and d) InGaN‐20 responding to NO_2_ over 60 days. T_1_ and T_2_ for 200 ppm of NO_2_ at RT and 30 % RH for the e) n‐GaN‐20 and f) InGaN‐20 responding over 60 days.

**Figure** **6** demonstrates the repeatability of the n‐GaN‐20 (Figure 6a) and InGaN‐20 (Figure 6b) through three response cycles, each consisting of at least four response‐recovery curves, measured at 100, 10, and 1 ppm NO_2_ gas concentrations under RT and 30% RH conditions. The results indicate stable response and recovery characteristics of the sensors. However, compared to conventional GaN‐based gas sensors, the sensors in this paper exhibit slightly inferior reproducibility, which can be primarily attributed to process variations in electrode fabrication and packaging. Adopting standard MEMS technology is expected to mitigate this issue effectively. The long‐term stability of the n‐GaN‐20 and InGaN‐20 were evaluated by monitoring its response value for 200 ppm NO_2_ continuously for 60 consecutive days, as depicted in Figure 6c–f. During this period, the response of InGaN‐20 to NO_2_ showed a gradual decline (Figure 6d) and the T_1_/T_2_ exhibited a gradual increase as the experiment proceeded. Conversely, the n‐GaN‐20 exhibited consistent performance with less than 9.19% response variation (Figure 6c). The T_1_ values showed tiny fluctuation and T_2_ desorption time progressively increased over the duration of the test (Figure 6e). Collectively, these observations demonstrate excellent long‐term stability and reliability for the n‐GaN‐20. During long‐term stability testing, prolonged environmental exposure leads to $O_{2}^{-}\left(\mathrm{ads}\right)$ accumulation on the sensor surface, reducing electron concentration. These adsorbed species limit active site availability for subsequent NO_2_ adsorption, resulting in diminished response amplitude, slower response kinetics, and prolonged recovery time.

**Table** **1** compares the performance of the proposed n‐GaN‐20 sensor and other previously reported GaN‐based NO_2_ gas sensors. The materials mentioned in Table 1 encompass GaN‐based materials, MOCVD‐synthesized materials, and other materials employed in NO_2_ detection. The n‐GaN‐20 exhibits high NO_2_ response, rapid T_1_/T_2_, and ultra‐low detection limit of 100 ppt. The LOD of NO_2_ for the n‐GaN‐20 and other reported NO_2_ gas sensors shown on Figure S13 (Supporting Information).

**Table 1:** Compares the performance of the proposed n‐GaN‐20 and other previously reported GaN‐based and NO_2_ gas sensors.

Material (MOCVD)	Detection gas	T [°C]	Concentration	Res. [%]	LOD	References/Year
ZnO/GaN (×)	NO_2_	125°C	2 ppm	134.5	20 ppb	^[^ ^32^ ^]^/2023
Ag‐In_2_O_3_ (√)	NO_2_	RT	5 ppm	59.7	140 ppb	^[^ ^33^ ^]^/2023
GaN/In_2_O_3_ (√)	NO_2_	100°C	100 ppb	238	1 ppb	^[^ ^34^ ^]^/2023
InGaN/GaN (√)	NO_2_	150°C	5 ppm	82.94	3 ppb	^[^ ^13^ ^]^/2024
MoSe_2_‐rGO (×)	NO_2_	RT	4 ppm	38	100 ppb	^[^ ^35^ ^]^/2025
Cu_3_(HIB)_2_ (×)	NO	RT	40 ppm	2500	1.8 ppb	^[^ ^36^ ^]^/2025
n‐GaN‐20 (√)	NO_2_	RT	200ppm	340	100 ppt	This paper

### 2.3 Gas Sensing Mechanisms

**Figure 7:**
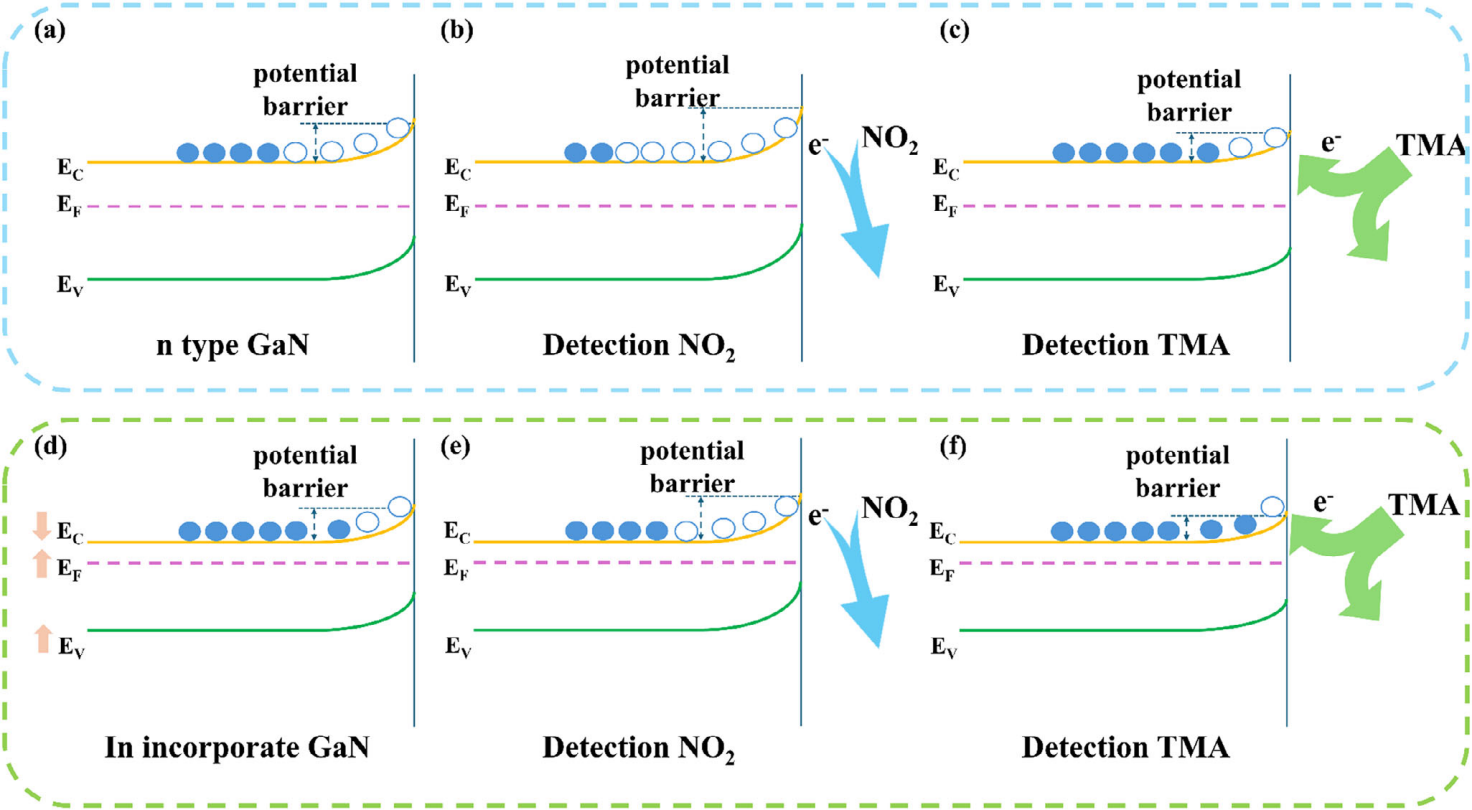
Schematic gas sensing mechanism of n‐GaN: a) in air, b) exposure to NO_2_ and d) exposure to TMA. Schematic gas sensing mechanism of InGaN: d) in air, e) exposure to NO_2_ and f) exposure to TMA.

With regard to the GaN materials, the incorporation of In component facilitates a shift in selectivity by modulating the band structure and increasing nitrogen vacancy concentration. The incorporation of In components causes a downward shift in the conduction band minimum (CBM) of surface GaN through the orbital contribution of In 5s electrons, while elevating the valance band maximum (VBM) through strengthened In‐N hybridization.^[^ ^37^, ^38^ ^]^ This synergistic effect results in substantial bandgap narrowing, which facilitates electron accumulation on the GaN surface and reduces the electron injection barrier (**Figure** **7**). Furthermore, In atoms substitution for Ga atoms induces lattice distortion due to the larger atomic radius of In atoms, generating nitrogen vacancies. Functioning as donor defects, nitrogen vacancies release electrons that shift the Fermi level toward the CBM, enhancing n‐type conductivity, while the resultant electron accumulation induces Fermi level pinning, stabilizing surface band bending and suppressing electron transfer to adsorbed oxygen. Consequently, the reduced electron transfer driving force^[^ ^39^ ^]^ ($\Delta E=E_{F}-E_{O_{2}}$) weakens oxygen adsorption capacity, lowering surface oxygen coverage. This cascade ultimately diminishes the GaN‐based sensor sensitivity toward oxidizing gases.^[^ ^40^ ^]^ In incorporation introduces shallow donor states, shifting the Fermi level toward the conduction band edge and enhancing n‐type conductivity. This change forms an energy level match with the low electron affinity energy of reducing gas, facilitating efficient electron transfer from gases to the conduction band and improving charge injection efficiency.^[^ ^41^ ^]^

**Figure 8:**
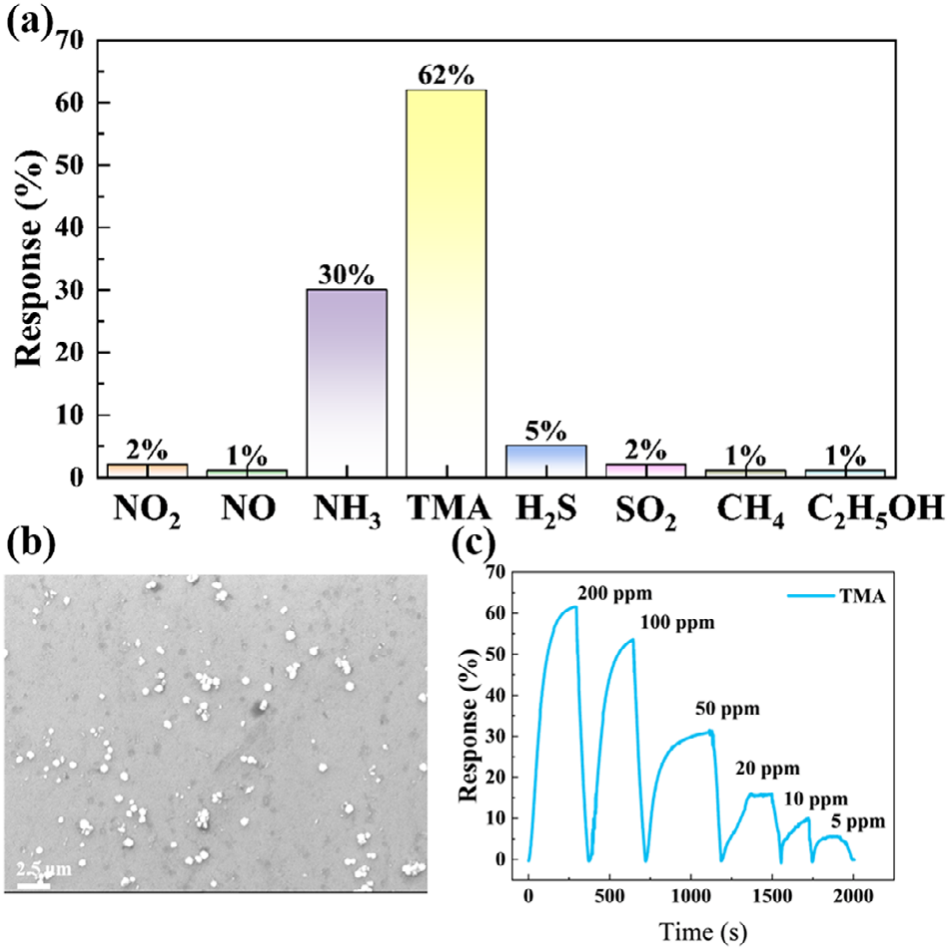
Surface topography and gas sensitivity performance of InGaN‐High. a) Selectivity of InGaN‐High. b) TEM of InGaN‐High. c) Dynamic response curves of InGaN‐High to 200–5 ppm TMA.

To investigate In incorporation effects on gas sensing, the high In content InGaN sensors were fabricated by increasing TMIn flow during MOCVD growth. The SEM images of the InGaN‐High is displayed as **Figure** **8**b, the spherical surface structures of In components can be observed, caused by the volatilization of In species due to the thermodynamic instability with high In concentration during MOCVD growth, while the detailed result of SEM and EDS is visible on Figure S14 (, Supporting Information). As depicted in Figure 8a, compared with the selectivity performance of the InGaN‐20 and InGaN‐15, the higher concentration of In composition significantly modulated the selectivity performance of the InGaN‐High, enhancing its responsiveness toward reducing gases. The dynamic response/recovery curves of the InGaN‐High in response to TMA concentrations from 200 to 5 ppm are illustrated in Figure 8c and Figure S15. The InGaN‐High exhibited a typical n‐type response to TMA gas and response decrease with the decreasing concentrations of TMA gas.

The n‐type semiconducting behavior of the InGaN‐High, InGaN‐15, InGaN‐20, and n‐GaN‐20 is confirmed by a decrease in resistance upon exposure to reducing gases and an increase in resistance under oxidizing gases. Figure S16 shows NO_2_ adsorption on the n‐GaN‐20/InGaN‐20 and TMA adsorption on the InGaN‐15/InGaN‐High across gas concentration gradients. The adsorption behavior was quantitatively assessed using the Langmuir isothermal adsorption model. The fitting result demonstrates that the mechanism of gas sensing for the n‐GaN‐20, InGaN‐20, InGaN‐15, and InGaN‐High is charging transfer. In n‐type semiconductor gas sensors, surface‐adsorbed oxygen forms reactive species. Oxidizing gases for NO_2_ and NO detection occurs through electron transfer from these oxygen species to oxidizing gases molecule. This electron depletion in the conduction band significantly increases electrical resistance. However, In incorporation impedes conduction band alignment with the electron affinity of NO_2_, reducing their response versus that of the n‐GaN‐20 at equivalent concentrations. Figure S18 displays the EPR spectra of vacancy of the sample of this work. All samples exhibit a symmetric derivative EPR peak (g ≈ 2.003), with intensity enhancement proportional to In concentration confirming increased vacancy concentration due to In incorporation.

**Figure 9:**
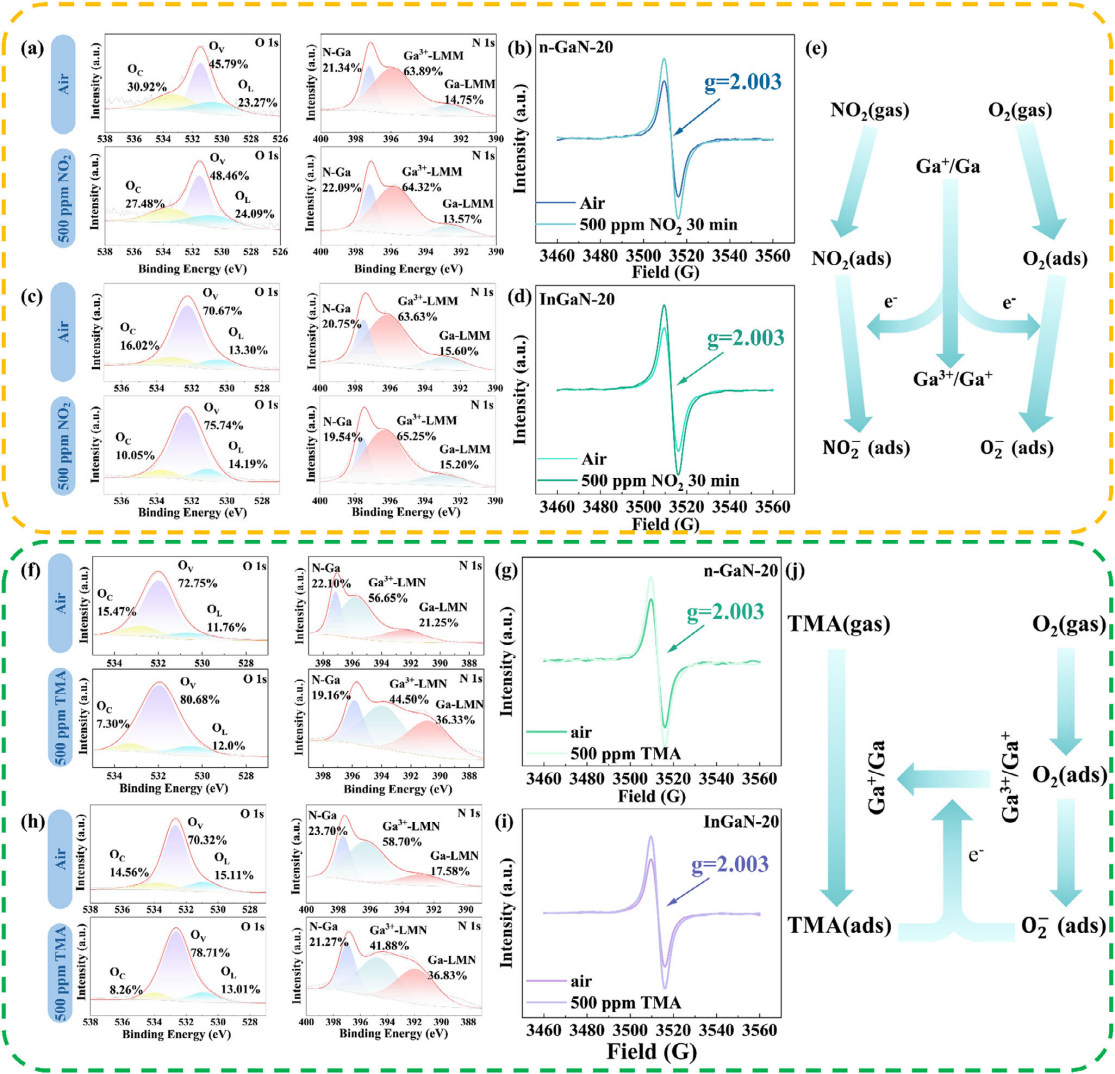
In situ characterization of the gas sensing mechanism of NO_2_ gas: a) In situ XPS, (b) In situ EPR monitoring under 500 ppm NO_2_ for 30 min for n‐GaN‐20. c) In situ XPS, d) In situ EPR monitoring under 500 ppm NO_2_ for 30 min of InGaN‐20. e) The state changes of the main elements in the NO_2_ detection. In situ characterization of the gas sensing mechanism of TMA gas: f) In situ XPS, g) In situ EPR monitoring under 500 ppm TMA for 30 min for n‐GaN‐20. h) In situ XPS, i) In situ EPR monitoring under 500 ppm TMA for 30 min for InGaN‐20. j) The state changes of the main elements in the TMA detection.

In situ monitoring of the sensors is essential for understanding the adsorption mechanism of oxidizing and reducing gases. To gain insight into the sensing mechanisms and interactions between oxidizing gas such as NO_2_ and the n‐GaN‐20 and InGaN‐20, In situ XPS analysis was conducted for further analysis upon continuous 500 ppm of NO_2_ exposure 30 min by capturing oxidative transformations during NO_2_ chemisorption. In the analysis of In situ XPS results, the evolution of the relative proportions of characteristic peaks was examined to investigate the gas sensing mechanism. As shown in the N 1s spectra of **Figure** **9**a,c, the intensified Ga^3+^‐LMM Auger peak confirms the occurrence of oxidation reaction, which can be attributed to the NO_2_ interaction with the GaN surface facilitates electron transfer to oxidize surface Ga atoms into Ga^3+^ species. Concurrently, surface oxygen species are consumed through NO_2_ chemisorption, releasing oxygen atoms that incorporate into the lattice as interstitial defects; this process increases lattice oxygen content while triggering charge compensation through oxygen vacancy formation, consistent with the enhanced of O_V_/O_L_ and attenuated O_C_ peaks in O 1s spectra of Figure 9a,c. Furthermore, In situ EPR analysis revealed vacancy evolution during NO_2_ chemisorption, as evidenced by a increase in g ≈ 2.003 signal intensity depicted on Figure 9b,d. The enhancement of signal intensity can be attributed to the increase of nitrogen vacancies. Nitrogen vacancy generation originates from surface‐adsorbed NO_2_ captures free electrons to form chemisorbed species which shifts the Fermi level toward VBM, weakens N‐lattice bonding and triggers nitrogen cation detachment for charge compensation, which will generate nitrogen vacancies. This mechanism is corroborated by In situ XPS showing decreased N 1s binding energy. The chemical reactions for these interactions can be represented as follows:

(2) $$O_{2\left(\mathrm{gas}\right)}\to O_{2\left(\mathrm{ads}\right)}$$

(3) $$O_{2\left(\mathrm{ads}\right)}+e^{-}\to O_{2\left(\mathrm{ads}\right)}^{-}$$

(4) $${\mathrm{NO}}_{2\left(\mathrm{gas}\right)}+e^{-}\to {\mathrm{NO}}_{2\left(\mathrm{ads}\right)}^{-}$$

(5) $${\mathrm{NO}}_{2\left(\mathrm{gas}\right)}+O_{2\left(\mathrm{ads}\right)}^{-}+2e^{-}\to {\mathrm{NO}}_{2\left(\mathrm{ads}\right)}^{-}+2O_{\left(\mathrm{ads}\right)}^{-}$$

(6) $${\mathrm{Ga}}_{\left(\mathrm{ads}\right)}^{+}/\mathrm{Ga}\to {\mathrm{Ga}}_{\left(\mathrm{ads}\right)}^{3+}/{\mathrm{Ga}}_{\left(\mathrm{ads}\right)}^{+}+3e^{-}$$

In situ XPS analysis under continuous 500 ppm TMA exposure for 30 min elucidated mechanistic insights into interactions between the reducing gas TMA and the n‐GaN‐20 and InGaN‐20. TMA acts as an electron donor, reducing surface Ga^3+^ species to lower oxidation states or metallic Ga through electron transfer. In situ XPS analysis results confirm this reduction through decreased Ga^3+^‐LMM Auger and increased Ga‐LMM Auger intensity as shown in N 1s spectra of Figure 9f,h. Moreover, the O 1s spectra of Figure 9f,h displays the attenuation of O_C_/O_L_ and enhanced of O_V_ peak, electron transfer reduces part of Ga^3+^ to lower oxidation states caused the decrease of the content of lattice oxygen. Charging balance is maintained through oxygen vacancies formation and lattice oxygen diffusion. Concurrently, the lone pairs of N in TMA coordinate with surface Ga atoms, creating electron‐deficient Ga sites evidenced by increased Ga XPS binding energy (Figures S15c and S16c, Supporting Information). The chemical reactions for these interactions can be represented as follows:

(7) $$4\mathrm{TMA}+21O_{2\left(\mathrm{ads}\right)}^{-}\to 2N_{2}+8H_{2}O+12CO_{2}+21O_{\;\left(\mathrm{ads}\right)}^{-}$$

(8) $${\mathrm{Ga}}_{\left(\mathrm{ads}\right)}^{3+}/{\mathrm{Ga}}_{\left(\mathrm{ads}\right)}^{+}+e^{-}\to {\mathrm{Ga}}_{\left(\mathrm{ads}\right)}^{+}/\mathrm{Ga}$$

In situ XPS results confirm higher Ga^3+^ reduction extent in the InGaN‐20 than that of the n‐GaN‐20, demonstrating In incorporation enhances reducing gas detection. Furthermore, In situ EPR analysis revealed vacancy evolution during TMA chemisorption, as evidenced by a increase in g ≈ 2.003 signal intensity shown on Figure 9g,i. The increase of signal intensity can be attributed to TMA injection supplying electrons to the GaN surface, thereby increasing the surface electron concentration, lowering the nitrogen vacancy formation energy though charge compensation, and promoting nitrogen lattice detachment and vacancy generation. Moreover, electron injections narrow the depletion layer, reducing its inhibitory effect on vacancy formation and further lowering nitrogen vacancies formation energy. Competitive binding at nitrogen lattice sites promotes displacement and detachment of lattice nitrogen atoms, generating additional nitrogen vacancies defects.

To sum up, The GaN‐based gas sensor developed in this work achieves tunable selectivity through the incorporation of an In component. This modification shifts the sensor's selectivity from detecting oxidizing gases (such as NO_2_ and NO) toward detecting reducing gases (including TMA and NH_3_). The XPS analysis revealed that the incorporation of In modulates the concentration of oxygen species within the material, and this modulation serves as the underlying mechanism for the observed alteration in gas selectivity. The XPS results demonstrate that the incorporation of In leads to an increase in oxygen vacancies and lattice oxygen content, while decreasing the adsorbed oxygen content. The elevated concentration of oxygen vacancies and lattice oxygen provides additional adsorption and reaction sites for reducing gas molecules. Concurrently, the increased oxygen vacancies donate more electrons to the conduction band of the GaN‐based material, thereby enhancing its baseline electrical conductivity. Reducing gas molecules (such as TMA and NH_3_) typically react with adsorbed oxygen or lattice oxygen on the material surface, releasing electrons into the material during this process. Consequently, upon exposure to reducing gases, these molecules inject electrons into the surface. Since the baseline conductivity is already elevated due to the increased oxygen vacancies, these additional electrons induce a more pronounced increase in the material's electrical conductivity. This amplified conductivity change translates to enhanced detectability. Furthermore, the increased oxygen vacancies provide additional reaction sites, synergistically facilitating this process and collectively contributing to the improved detection capability. Regarding oxidizing gas molecules (such as NO_2_ and NO), they tend to extract electrons from the material, thereby reducing its electrical conductivity. This process typically relies on pre‐adsorbed oxygen species on the material surface. However, the incorporation of In reduces the content of adsorbed oxygen, leading to a decrease in the number of active sites available for electron exchange with oxidizing gases. Consequently, when oxidizing gases are present, fewer electrons can be captured by these gases, resulting in a diminished detection response. Thus, the reduced availability of electron‐accepting sites directly contributes to the weakened sensing capability toward oxidizing gases.

### 2.4 Disease Monitoring Through Exhaled Breath

**Figure 10:**
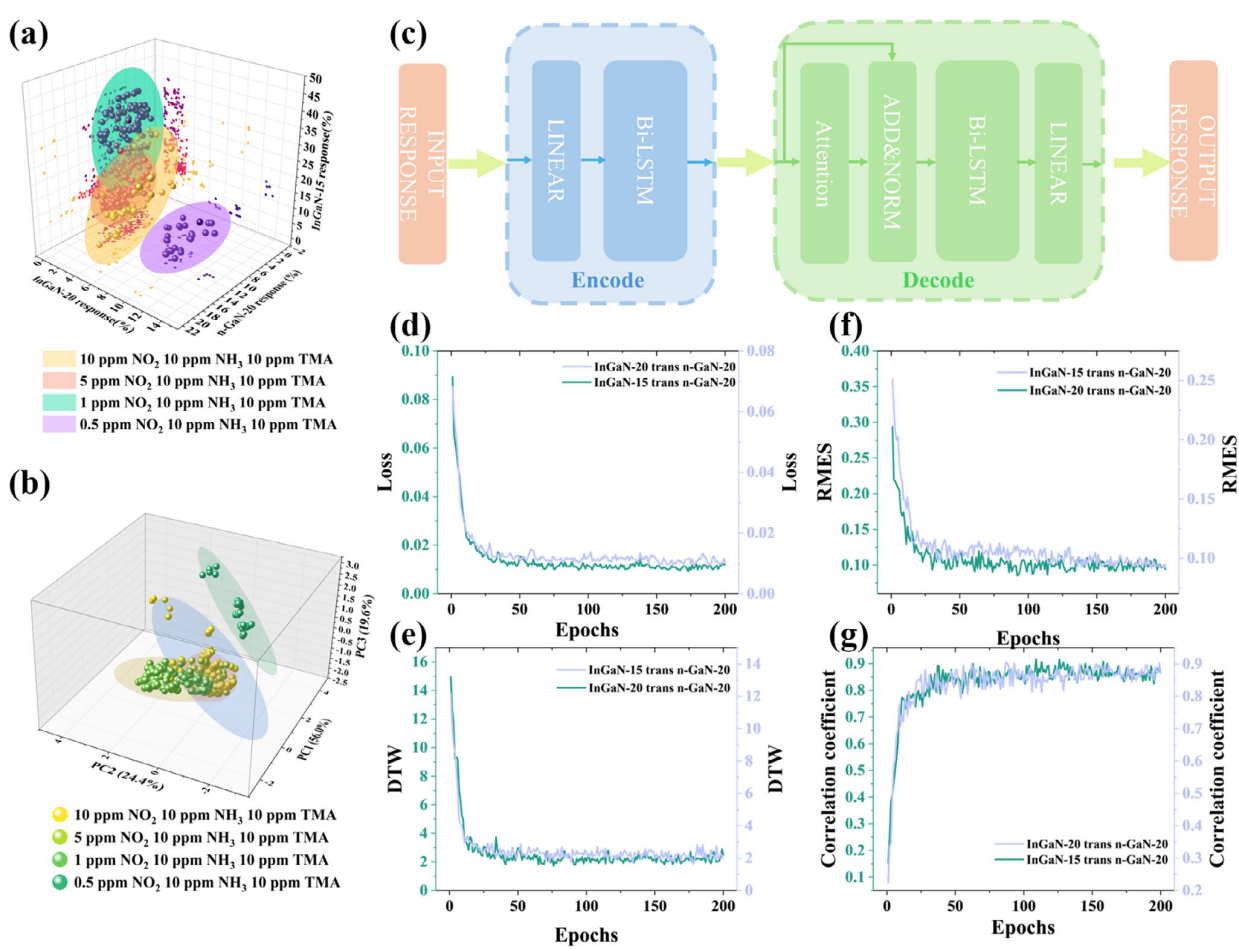
a) original response data distribution on 3D. b) PCA reconstructed data distribution on 3D. c) construction of mapping model. d) model Loss results. e) model DTW results. f) model RMSE results. g) model correlation coefficient results.

With the development of smart gas sensors, two fundamental constraints in sensor‐based intelligent systems are exposed. Firstly, Sensor‐specific response characteristics inherently limit cross‐sensor data interoperability, contrasting with the intrinsic data universality of large language models (LLMs) and computer vision (CV), thereby confining datasets and analytical models to closed ecosystems. Secondly, functional interdependencies between sensor data and models restrict cross‐sensor compatibility, with pronounced implications in high‐risk environments, disease detection, and irreplicable scenarios. These dual limitations necessitate sensor‐agnostic AI frameworks to enable scalable system integration. To achieve model generalization and data reusability based same device, this work proposes a unified data mapping preprocessing approach. By mapping the sensors response curve to other sensors response curve to achieve trained model generalization. To build the mapping model, a mix gas data set has been built with different concentrations of NO_2_, NH_3,_ and TMA which has good response for the sensors built in this paper. The distribution of response value of the InGaN‐15, InGaN‐20, and n‐GaN‐20 for mixture gases of different components is illustrated as **Figure** **10**a. The data demonstrates distinct clustering tendencies in different data categories. Furthermore, PCA feature extraction significantly enhances inter category separation as shown in Figure 10b, confirming differential response patterns across gas types.

This work built an Encoder‐Decoder model (Figure 10c) to map different types of sensors response curve. The input is the response curve of InGaN‐15 or InGaN‐20 for mixture gas condition, the output is the corresponding response of n‐GaN‐20 on same condition. To evaluate the model performance, this paper employs three quantitative metrics: RMSE, correlation coefficient and Dynamic Time Warping (DTW), the result depicted on Figure 10d–g. These metrics are used to systematically compare the mapping responses generated by the InGaN‐15 and InGaN‐20 response curves through models with the response curve of n‐GaN‐20 under same mixed‐gas conditions. After 200 training epochs, the loss score, RMSE, and correlation coefficient prove that the model successfully converged. Furthermore, this work used DTW to assess the generated outputs, the result demonstrates minimal discrepancies between the mapping response curves and the real response curve.

**Figure 11:**
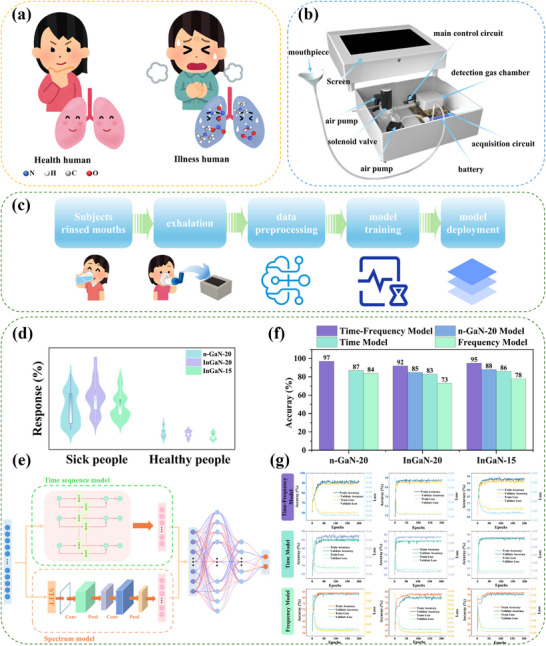
a) Differences in pulmonary gas composition between healthy individuals and patients. b) Composition of two‐channel exhaled breath collection device. c) Schematic diagram of Exhaled breath test. d) Comparison of response between healthy and diseased people. e) model train result and ablation result. f) structure of Time‐Spectrum model. g) model accuracy and loss result of each sensor.

With the development of medical detection, point‐of‐care testing (POCT), employing portable analytical instruments and reagents, enables rapid on‐site analysis that facilitates early disease diagnosis.^[^ ^42^ ^]^ With growing recognition of gaseous biomarkers for disease detection, gas sensor‐based POCT platforms offer noninvasive, rapid health assessment. The developed sensor in this paper demonstrates high NO_X_ detection sensitivity, indicating potential for lung disease diagnostics. To facilitate the efficient collection of human exhaled breath for disease diagnosis, the two‐channel exhaled breath collection device has been designed in this study (**Figure** **11**b). The composition of the two‐channel exhaled breath collection device is shown as Figures S22–S24 (Supporting Information). The testing procedure, as depicted in Figure 11c, involves participants performing an oral rinse followed by exhalation testing with concurrent signal acquisition for dataset construction, exhaled breath data were systematically collected from both healthy individuals and lung cancer patients. Specifically, the dataset comprises breath samples from six lung cancer patients and six healthy controls, with each participant undergoing fasting breath tests on six consecutive mornings to ensure temporal consistency and metabolic stability during data acquisition. As shown on Figure 11d, variations in biomarker concentrations across patient cohorts, attributable to differing disease progression stages, resulted in statistically significant inter‐group disparities.

In this study, we employ the STFT to convert sensor time series signals into spectrograms as part of the model input. A Time Frequency Analysis Model is developed in this paper for disease diagnosis. The model uses Bi‐LSTM to analyze the time sequence data and use CNN to analyze frequency data, finally, feature fusion is carried out, and an ANN network is constructed to predict results, the model struct is depicted as Figure 11d. In order to evaluate the performance of the model, ablation experiments are conducted to systematically compare the diagnostic capabilities of standalone time sequence models and spectrogram models. To enhance model convergence and generalization capability, this paper uses a cosine annealing strategy for learning rate scheduling, with cyclical adjustments executed every 20 epochs. The model training results and ablation experimental outcomes are presented in Figure 11e. The model exhibits effective identification capabilities for exhaled breath diseases, achieving recognition accuracy of 97%, 92% and 95% when trained on Dataset of n‐GaN‐20, InGaN‐20, and InGaN‐15 response data, respectively. Ablation experiment results indicate that models constructed using single data types perform slightly inferior to the proposed joint model in this study (Figure 11e). The accuracy, recall, and F1 score of each model for the three sets of sensors is shown in Tables S4 and S5 (Supporting Information). To further validate the capability of the previously developed Encoder‐Decoder mapping model, exhaled breath responses of InGaN‐20 and InGaN‐15 were mapped to corresponding responses of n‐GaN‐20 using Encoder‐Decoder mapping model. Then use the Time Frequency Analysis Model to analyze the mapping data, the results demonstrate that the accuracy of disease recognition using mapped data with n‐GaN‐20's identification model is slightly lower compared to direct use the self‐models, yet the accuracy still surpasses the performance of standalone single models. This result validates that the mapping approach in this paper enables effective model generalization. However, the recognition accuracy of the model still needs further optimization. Morever, the constructed exhaled breath collection system exhibits generality, enabling multi‐disease diagnostics through interchangeable sensor configurations targeting distinct biomarkers.

## 3 Conclusion

This work proposes a controllable fabrication method for high‐performance gas sensors. The n‐GaN‐20 sensor exhibited exceptional sensitivity with ultra‐low detection limits of 100 ppt for NO_2_ and 2 ppb for NO. Furthermore, the incorporation of In components enabled selective modulation, with the InGaN sensors demonstrating the shift trend of selective to reducing gas gases. Mechanistic analysis through In situ characterization techniques revealed a synergistic lattice oxygen‐adsorbed oxygen regulation mechanism by incorporation In component to change the selective of sensors. To address multi‐sensor compatibility, a response signal mapping model was trained with mixed gas data, improving the generalization recognition algorithms. Furthermore, a dual‐channel breath analysis platform integrating the sensor array and a time‐sequence model was constructed, enabling non‐invasive disease diagnosis through exhaled biomarker profiling. The exhaled breath collection system constructed in this study exhibits considerable versatility. By changing the sensor array, the system can be adapted for the detection of various biomarkers, thereby facilitating its application in the diagnosis of multiple diseases. This work advances gas sensor design by linking material engineering, mechanistic insights, and system‐level integration, offering promising applications in precision healthcare.

## 4 Experimental Section

### Preparation of wafer

This study used MOCVD to produce the GaN‐based film sensors. The schematic of the sensor preparation process is presented in Figure S1 (Supporting Information).

### Characterization

The prepared composite GaN was investigated using various characterization methods. The characterization equipment used is listed in Table S1 (Supporting Information). The gas sensing properties of the fabricated sensors were evaluated using a CGS‐MT intelligent gas sensing analysis system with 5 L volume gas chamber under ambient conditions at room temperature and 30% relative humidity.

### Informed Consent

This study obtained written informed consent from all six patients and six healthy subjects.

## Conflict of Interest

The authors declare no conflict of interest.

## Supporting information



Supporting Information

## Data Availability

The data that support the findings of this study are available from the corresponding author upon reasonable request.
