# Supplementary material for: Oxygen‐Regulated GaN‐Based Sensors Fabricated by MOCVD for Switchable Gas Detection: Exhaled Gas Smart Platform for Non‐Invasive Disease Detection
Source: Adv Sci (Weinh). 2025 Nov 19;13(7):e15282. doi: 10.1002/advs.202515282 (PMC12866776; doi:10.1002/advs.202515282)
Supplement: Supplementary file 1 — Supporting Information [file ADVS-13-e15282-s001.docx]

Supporting Information

**Oxygen-Regulated GaN-based Sensors fabricated by MOCVD for Switchable Gas Detection: Exhaled Gas Smart Platform for Non-Invasive·Disease Detection**

Yuxuan Wang †, Dan Han ^*^ †, Qi Duan, Zhekai Zhang, Zhengyang Jia, Juxu Guang, Guojing Wang, Weidong Wang, Xiuli He, Shengbo Sang ^*^


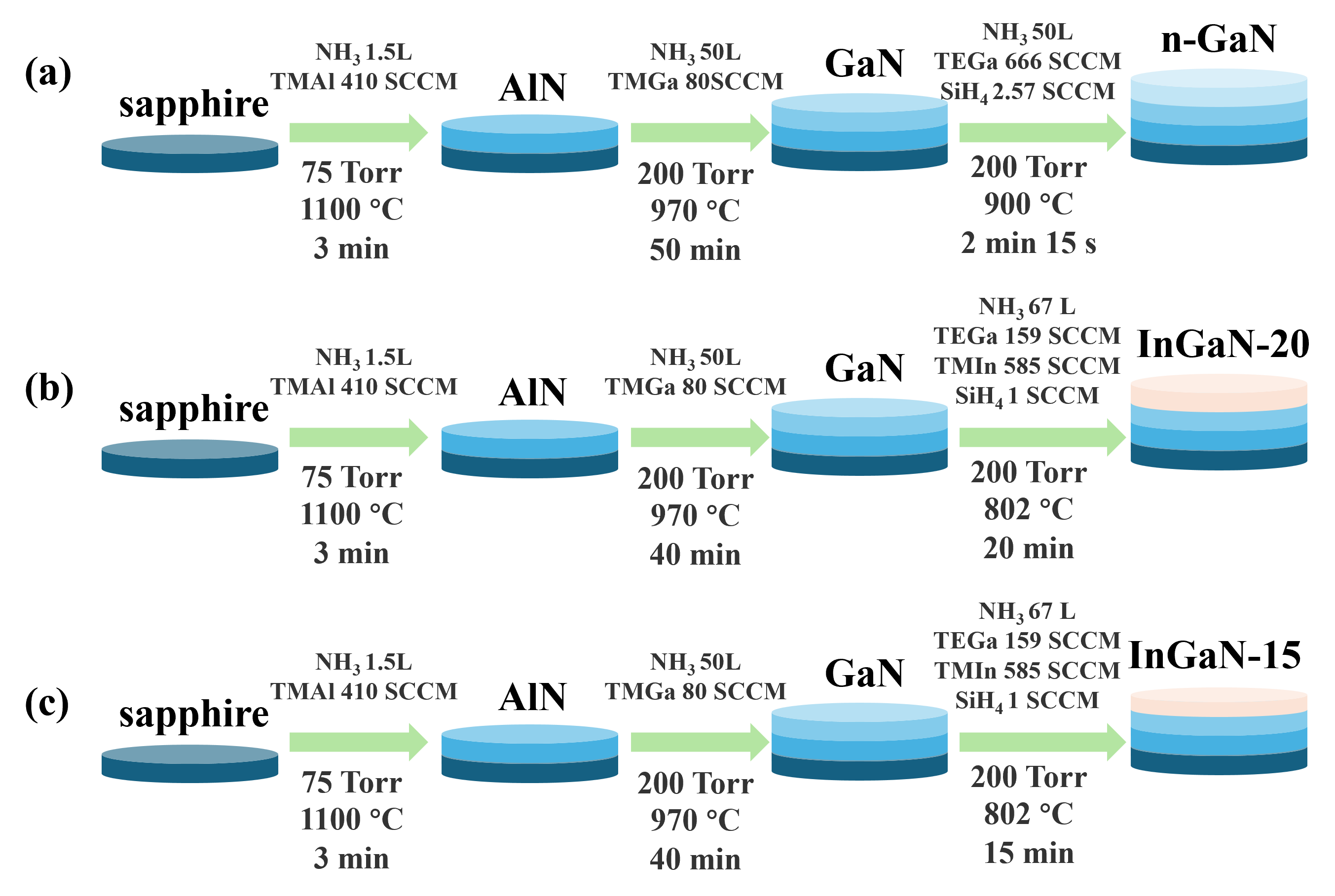


**Supplementary Figure 1.** Schematic of the preparation process of gas sensors.

**Supplementary Note 1:** The GaN based film sensors Firstly, an AlN buffer layer was grown on a sapphire substrate under conditions with 1100°C and 75 Torr for 3 minutes. The flow of NH_3_ and TMAl were 1.5 slm and 410 sccm, respectively. Secondly, the GaN epitaxial layer was grown by changing the reaction temperature to 970°C and increasing the pressure to 200 Torr. For 50 minutes growth phase, NH_3_ and TMGa were maintained at flow rates of 50 slm and 80 sccm, respectively, resulting in high quality GaN single crystalline formation. The final stage involved constructing the n-type doped layer at 900°C and 200 Torr, where NH_3_ and TEGa flows were 50 slm and 666 sccm. This growth process used SiH_4_ as the dopant source. Finally obtained the n-Ga-20 film. Furthermore, the InGaN-20 film was built by incorporating In using TMIn as the precursor on the n-GaN-20, aiming to investigate the effect of incorporation of In composition on sensing performance. Moreover, the InGaN-15 film with decrease the thickness of film was built by reducing film growth time, enabling analysis the effect of the thickness on sensing performance. In order to get the GaN based sensors, the prepared films were first cleaned. After cleaning, the Ti (30 nm)/Ni (80 nm)/Au (200 nm) multilayer electrodes were sequentially deposited through magnetron sputtering. Finally, the wafer was diced into individual sensors.


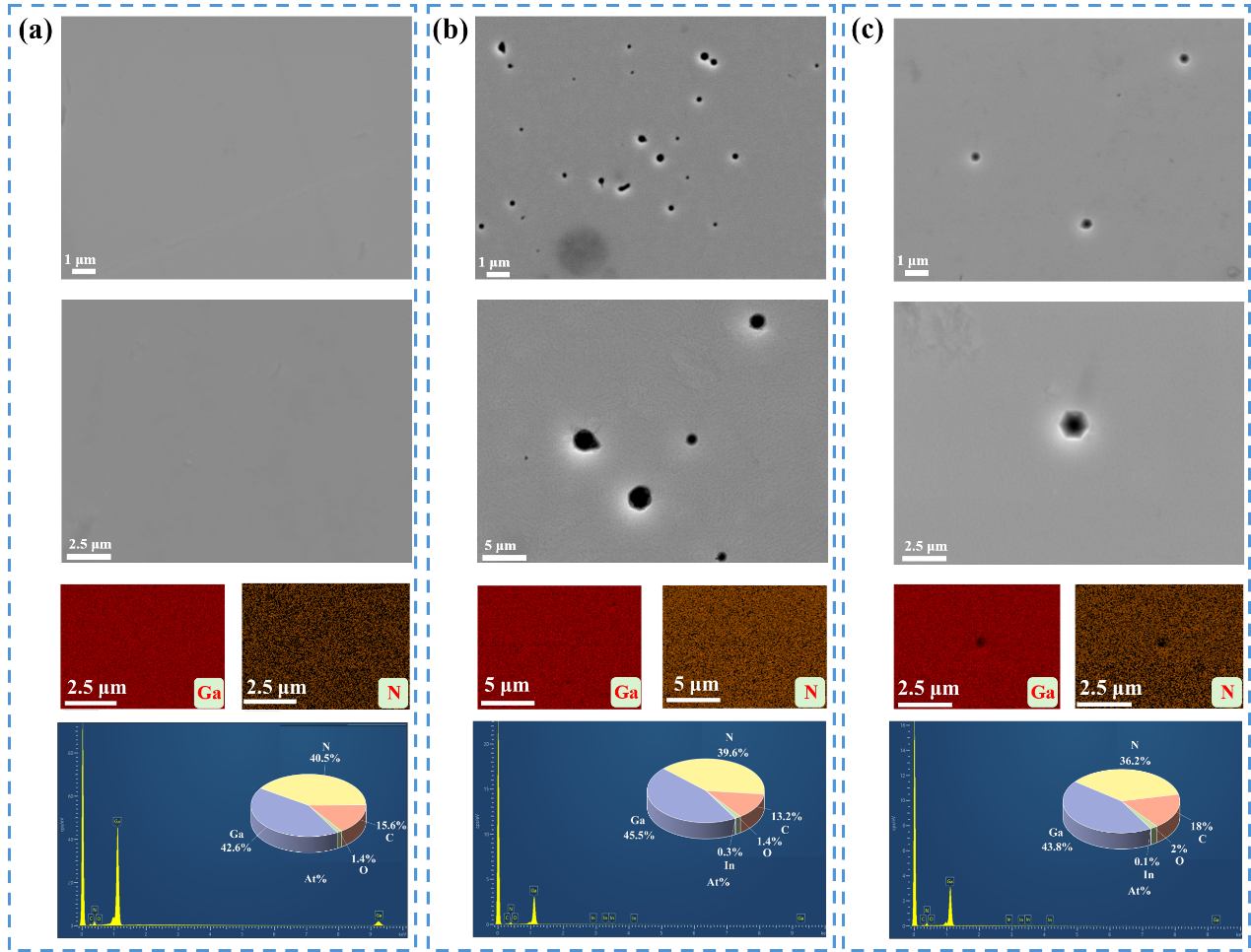


**Supplementary Figure 2**. SEM and EDS images results of the (a) n-GaN-20, (b) InGaN-20, (c) InGaN-15.

**Supplementary Note 2:** Supplementary Figure 2 depicts the top views of the n-GaN-20 (Supplementary Figure 2a), InGaN-20 (Supplementary Figure 2b) and InGaN-15 (Supplementary Figure 2c) sensors measured by SEM. Hexagonal pits were observed on the surfaces of InGaN-20 and InGaN-15, whereas the n-GaN-20 surface appeared flat. The difference in lattice parameters (11%) between GaN and InN results in a large lattice mismatch, inducing lattice distortion and consequently generating stress. To minimize strain energy, the system releases stress through defect formation, such as the hexagonal pit defects observed in InGaN-15 and InGaN-20. Moreover, the thicker cap layer in InGaN-20 induces greater stress compared to InGaN-15, resulting in the formation of more defects. To analysis of defect occurrences between the InGaN-15 and InGaN-20, defect densities were calculated. The calculated results show that the defect density of the InGaN-15 and InGaN-20 is 0.02 per/μm^2^ and 0.15 per/μm^2^, respectively. Furthermore, the hexagonal pit structures significantly increase the specific surface area, enhancing environmental contact. Supplementary Figure 2 also shows the EDS of the n-GaN-20, InGaN-20 and InGaN-15, revealing a Ga/N atom ratio of approximately 1:1.


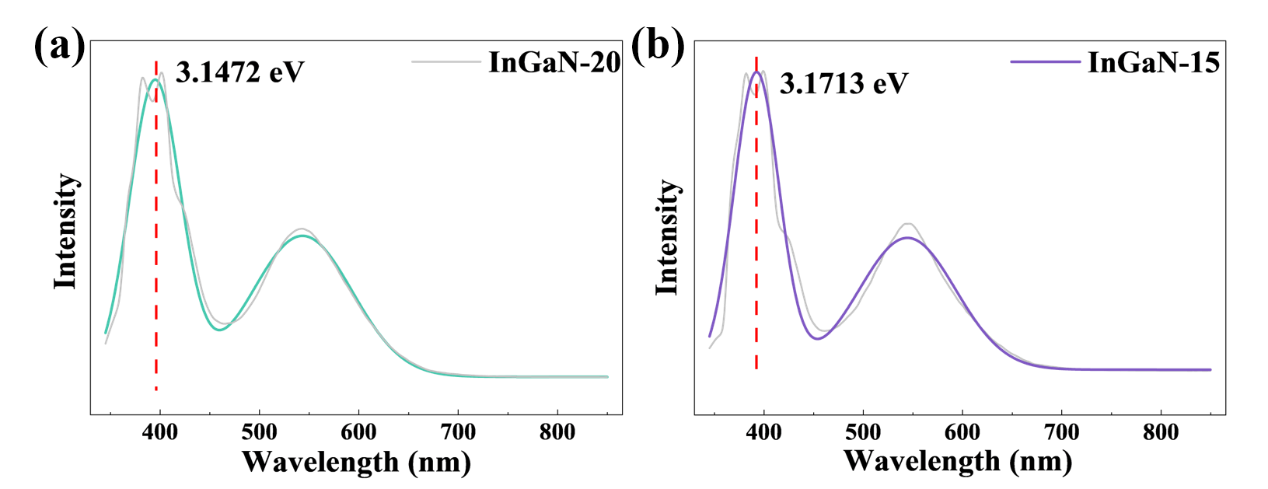


**Supplementary Figure 3**. PL curves of the (a) InGaN-20, (c) InGaN-15.

**Supplementary Note 3:** To determine the In composition in the fabricated samples, photoluminescence (PL) spectroscopy was employed for analysis. The corresponding PL spectrum is presented in the Supplementary Figure 3. Distinct emission peaks originating from the InGaN layer are observed in the spectra of the InGaN-20 and InGaN-15 samples, with peak energies measured at 3.1472 eV and 3.1713 eV, respectively. The peak energies for the InGaN-related emission are measured at 3.1472 eV and 3.1713 eV for the InGaN-20 and InGaN-15 samples, respectively. The In composition can be obtained from the Vegard law:

$$\begin{aligned} E_{g}^{{In}_{x}{Ga}_{1-x}N}=\left( 1-x \right)E_{g}^{GaN}+xE_{g}^{InN}-bx\left( 1-x \right)\#\left( 1 \right) \end{aligned}$$

where b is thxle bowing factor and is set as 1.43 eV. The In composition obtained by formula is 6.256% for InGaN-20 and 5.648% for InGaN-15. The test results indicate that the In composition in the InGaN-20 and InGaN-15 is similar; however, differing growth times led to variations in film thickness, which consequently affected their structural characterization and gas sensing performance.


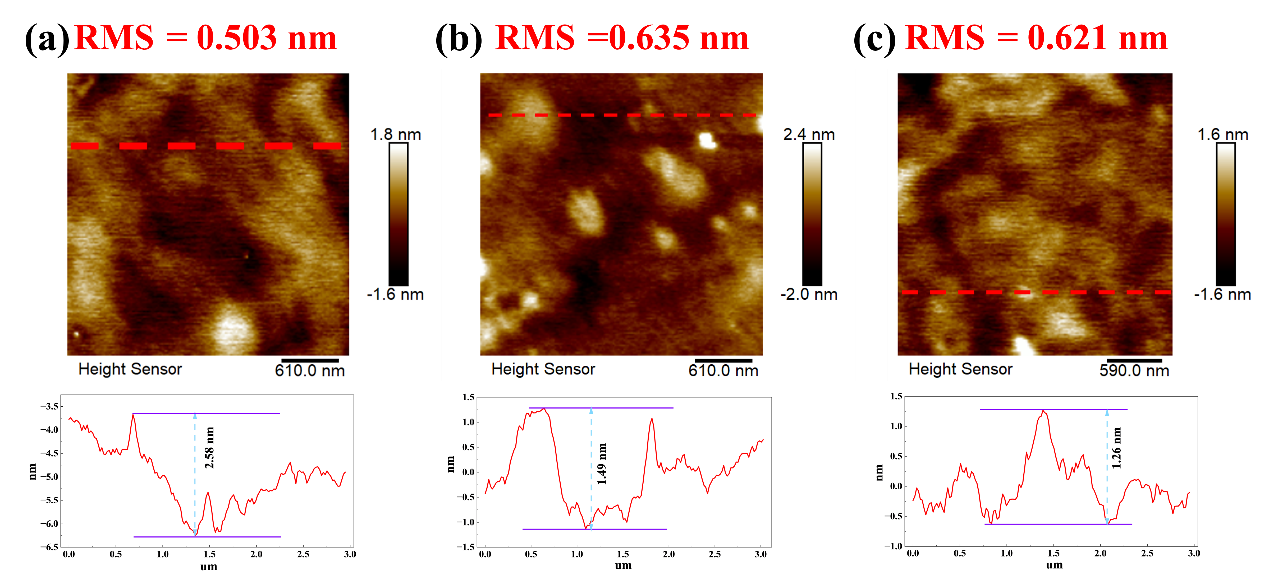


**Supplementary Figure 4**. AFM 2D images results of the (a) n-GaN-20, (b) InGaN-20, (c) InGaN-15.

**Supplementary Note 4:** The surface morphology and roughness of the GaN-based films were analyzed using atomic force microscopy (AFM) in tapping mode over a 3 μm × 3 μm scan area. The 2D AFM images of the n-GaN-20, InGaN-20 and InGaN-15 shown in Supplementary Figure 4, the test result reveal films with relatively smooth features, exhibiting a root mean square (RMS) roughness of approximately 0.503 nm, 0.635 nm and 0.621 nm, respectively. The measurements reveal an increase in the surface roughness of the thin film with the incorporation of In content. Furthermore, extending the growth time in the MOCVD process was also observed to result in a higher surface roughness. The increased surface roughness in films are expected to enhance gas adsorption by providing a larger active surface area and more adsorption sites, thereby contributing to its improved gas sensing performance.


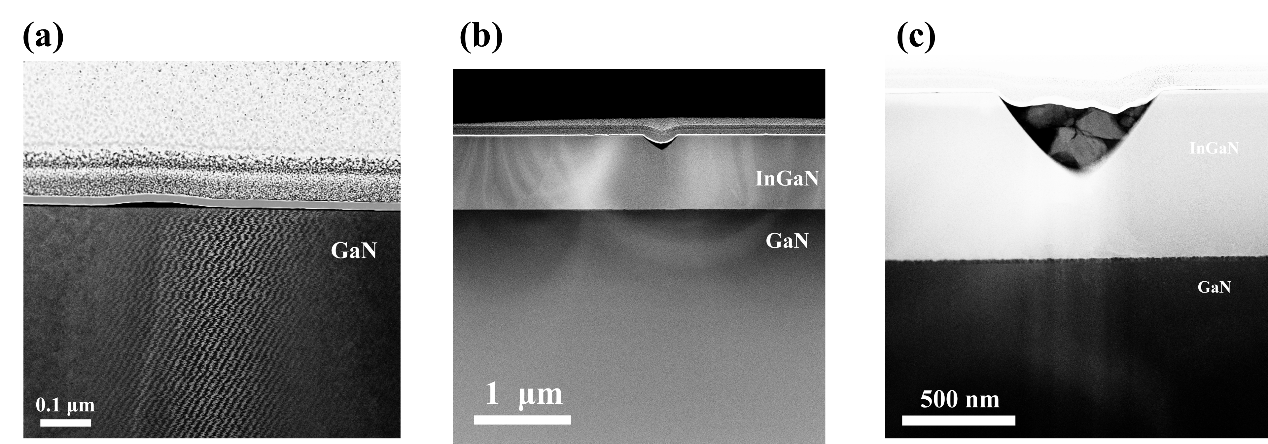


**Supplementary Figure 5**. TEM analysis of cross-sectional views of the (a) n-GaN-20, (b) InGaN-20 and (c) InGaN-15.

**Supplementary Note 5:** The cross-sectional morphology of n-GaN-20, InGaN-20 and InGaN-15 exhibited distinct layering, consistent with the preparation objectives.


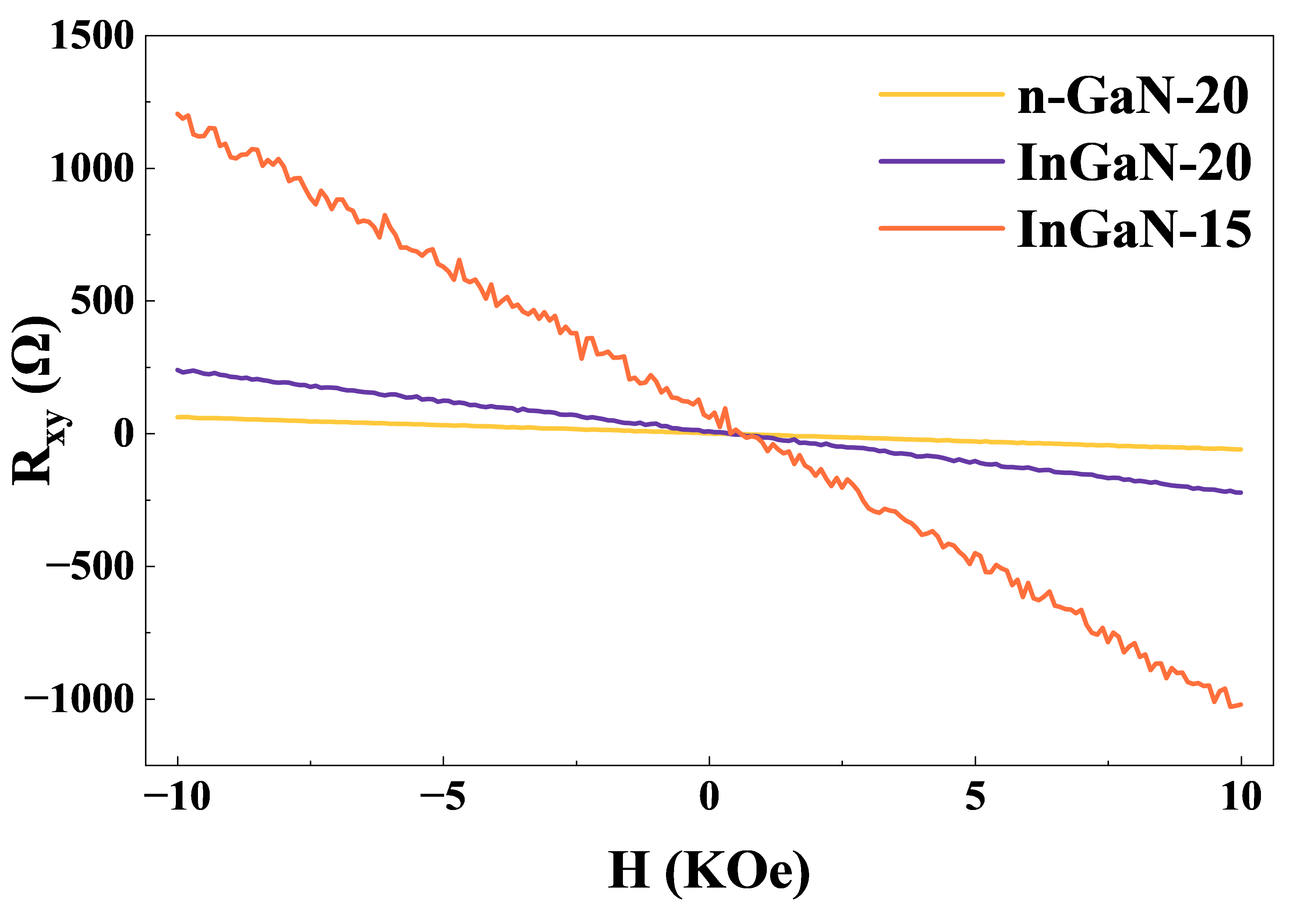


**Supplementary Figure 6**. Hall effect results obtained using the Van der Pauw method for the n-GaN-20, InGaN-20 and InGaN-20.

**Supplementary Note 6:** To determine the carrier mobility of the prepared materials, the van der Pauw method was employed in this paper to measure the Hall effect for calculating the carrier mobility. As shown in Supplementary Figure 6, the test results exhibit a negative slope, confirming that the majority carriers are n-type. Furthermore, the good linear relationship indicates that the electrical transport behavior of the material follows the classical single-carrier model. A larger absolute value of the slope of the test curve suggests a lower carrier concentration. The calculated carrier concentration and carrier mobility of the material are summarized in Supplementary Table 2. The results demonstrate that the incorporation of In components leads to a reduction in carrier mobility, which is attributed to the introduction of additional grain boundary barrier interfaces.


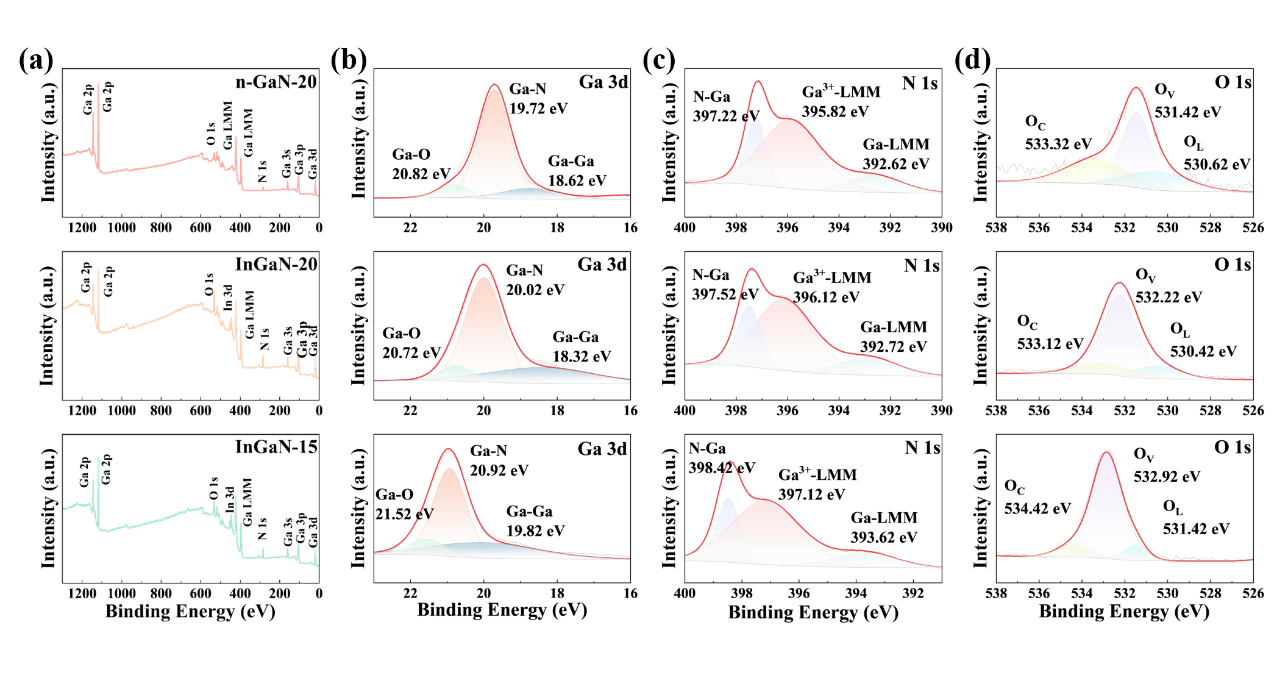


**Supplementary Figure 7**. (a)The overall XPS survey spectrum; XPS spectra of (b) Ga 3d, (c) N 1s and (d) O 1s of InGaN-15, InGaN-20, n-GaN-20

**Supplementary Note 7:** To investigate the elemental composition and surface chemical bonding of materials, XPS analysis was conducted. The overall XPS survey spectrum confirms that the constituent elements of n-GaN-20 are Ga, N, O, and C, and for InGaN-20 and InGaN-15 are Ga, In, N, O, and C, with no detectable impurity elements (Supplementary Figure 7a). As shown in Supplementary Figure 7b, The Ga 3d peak of GaN film was deconvoluted into three sub-peaks located at approximately 21.0 eV, 19.9 eV, and 18.9 eV, corresponding to Ga-Ga, Ga-N, and Ga-O bonding, respectively ^[1]^. The results indicate that the binding energy of the Ga 3d peaks for both InGaN-15 and InGaN-20 shift to higher values, suggesting a reduction in electron density around Ga atoms. This phenomenon can be attributed to an increase in surface Lewis acid sites which enhances the detection of reducing gases. The N 1s peak, as depicted in Supplementary Figure 7c, exhibits three distinct peaks centered at binding energies of 398.3 and 397 eV and 392.5 eV, attributable to N-Ga and Ga^3+^-LMM and Ga-LMM, respectively ^[2]^. The O 1s peak (Supplementary Figure 7d) can be deconvoluted into three peaks at 530, 531 and 533.3 eV, corresponding to lattice oxygen (O_L_), oxygen vacancy (O_V_) and chemisorbed oxygen (O_C_) ^[3]^, which is consistent with values reported in the cited literature. The lowest O_L_ content in n-GaN-20 indicates higher lattice order and crystallinity. Compared to n-GaN-20, the InGaN-15 and InGaN-20 have more O_L_ which contributes to the incorporation of the In component. The In atoms will replace the Ga atoms in the GaN lattice, the bigger atoms radius will induce lattice strain. The strain provides the access for oxygen atoms to incorporate into the GaN lattice, ultimately leading to an increase in the concentration of O_L_. Due to the thickest InGaN film, InGaN-20 exhibits the highest defect density, as manifested by the greatest amount of O_L_. As the O_L_ content increases, the intensity of the O_C_ peak in XPS spectra decreases, indicating a reduction in adsorbed oxygen content. The n-GaN-20 exhibits the highest O_C_ content; more O_C_ can contribute to the electron transfer process and provide active sites for the interaction with target gas molecules in gas sensors. The lower O_C_ content in InGaN-20 consistent with its higher defect densities, as confirmed by TEM analysis. Furthermore, compared to InGaN-20 and n-GaN-20, the peaks of InGaN-15 shift to higher energy, which means the InGaN-15 is harder to lose the electrons. Furthermore, a noticeable broadening of the O_V_ peak was observed in the InGaN-20 and InGaN-15. The incorporation of In induces heterogeneity in the bonding environment, leading to the observed O_V_ peak broadening. The electronegativity and atomic radius differences between In and Ga lead to variations in In-O and Ga-O bond lengths and energies, diversifying the local chemical environment of oxygen vacancies and consequently widening the binding energy distribution in XPS.


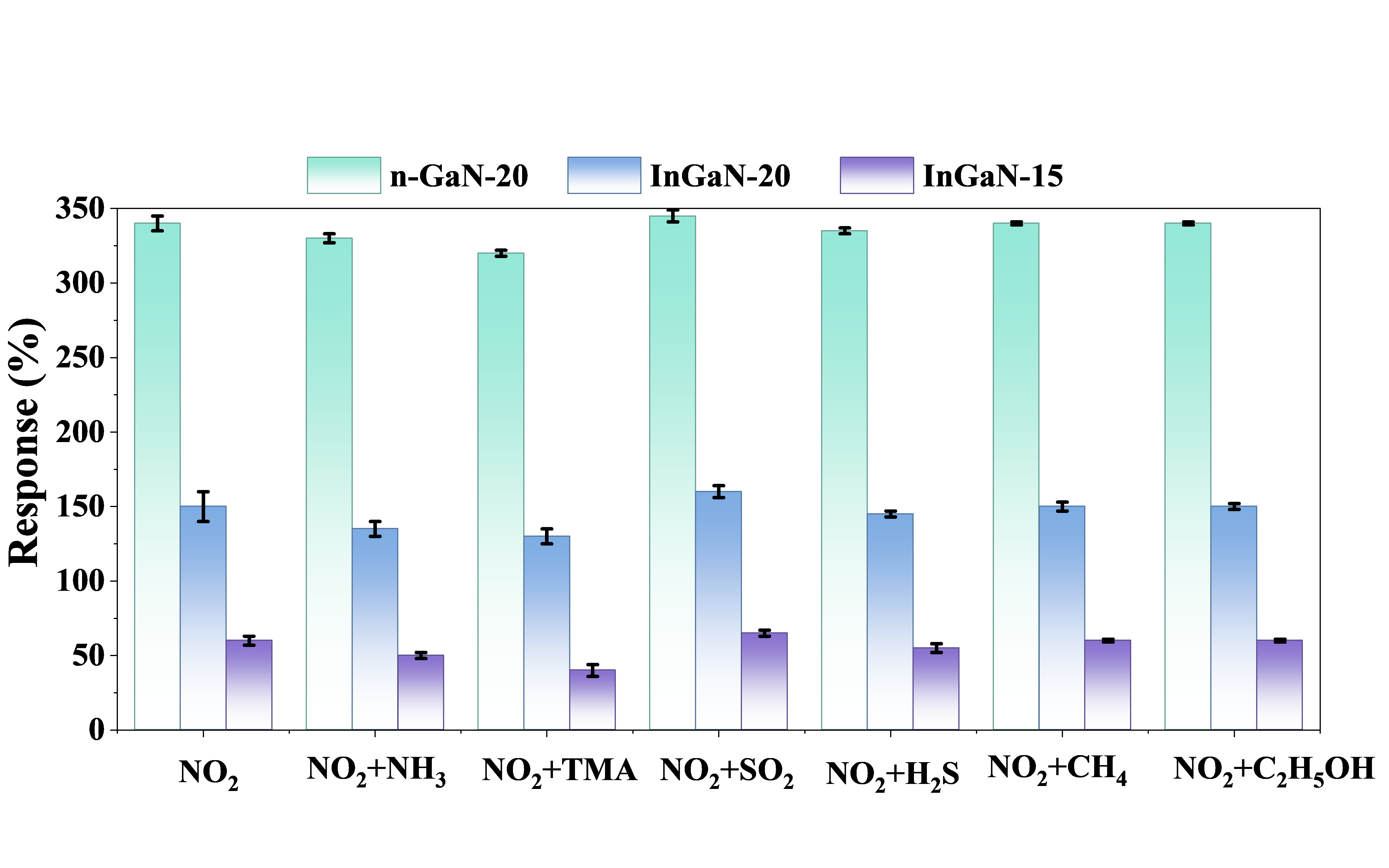


**Supplementary Figure 8**. Anti-interference capability of the n-GaN-20, InGaN-15 and InGaN-20.

**Supplementary Note 8:** To evaluate the anti-interference capability of the fabricated sensors, their responses to mixtures of 200 ppm NO_2_ and 200 ppm of six different gases (NH_3_, TMA, SO_2_, H_2_S, CH_4_, and C_2_H_5_OH) were tested. Each gas mixture was measured three times, and the average value was taken as the result. The findings indicate that the sensors generally exhibit good anti-interference performance. However, a slight decrease in response was observed during the tests involving mixtures with NH_3_ and TMA. Specifically, the InGaN-20 and InGaN-15 sensors experienced more significant interference. The average response decrease for n-GaN-20 was 10% and 15% in the presence of NH_3_ and TMA mixtures, respectively; for InGaN-20, the average decrease was 15% and 17%; and for InGaN-15, it was 10% and 20%. This heightened susceptibility is likely attributable to the InGaN-20 and InGaN-15 has greater sensitivity toward reducing gases.


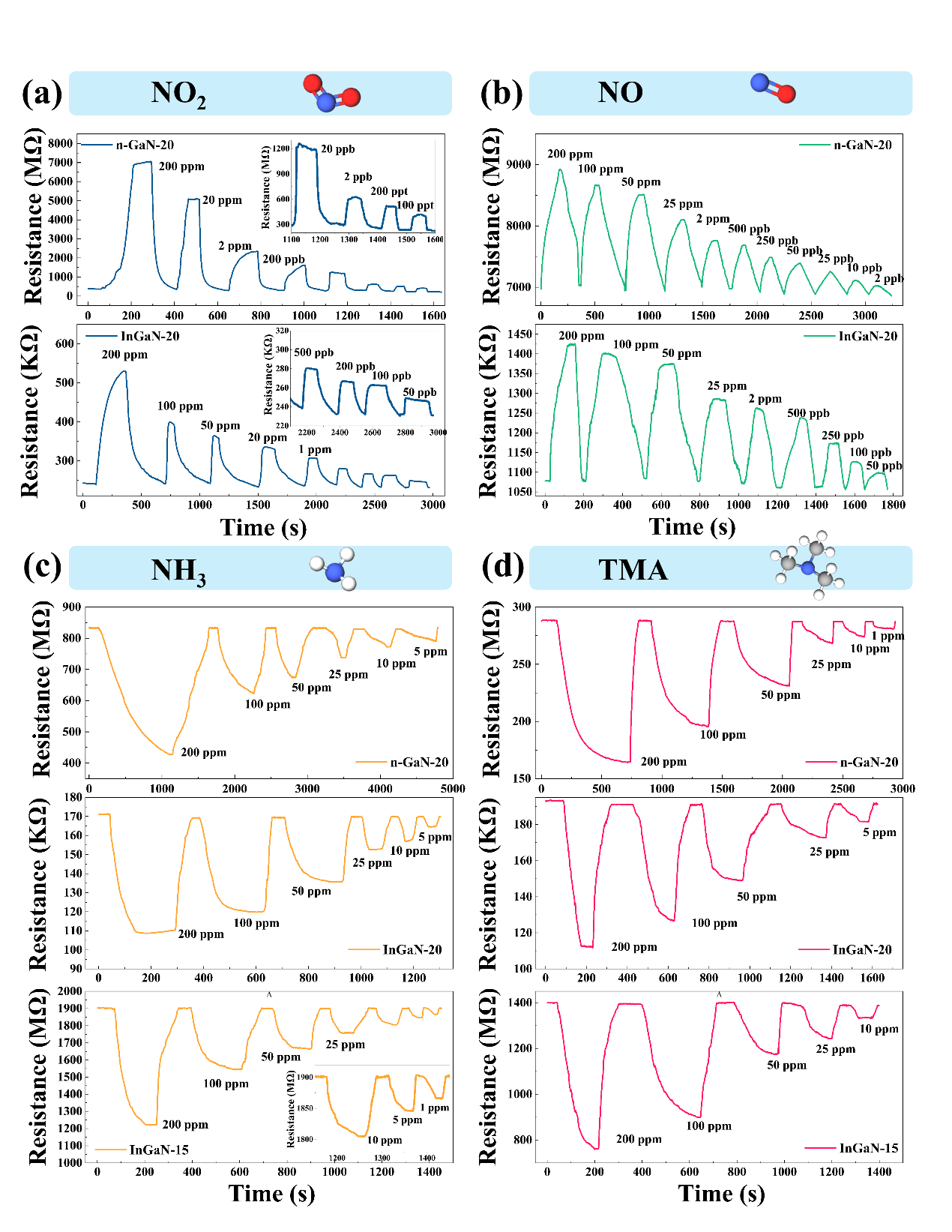


**Supplementary Figure 9**. Resistance curves of n-GaN-20, InGaN-20 sensors for Oxidizing gas: (a) NO_2_, (b) NO. Resistance curves of n-GaN-20, InGaN-20 and InGaN-15 sensors for Reducing gas: (c) NH_3_, (d) TMA.


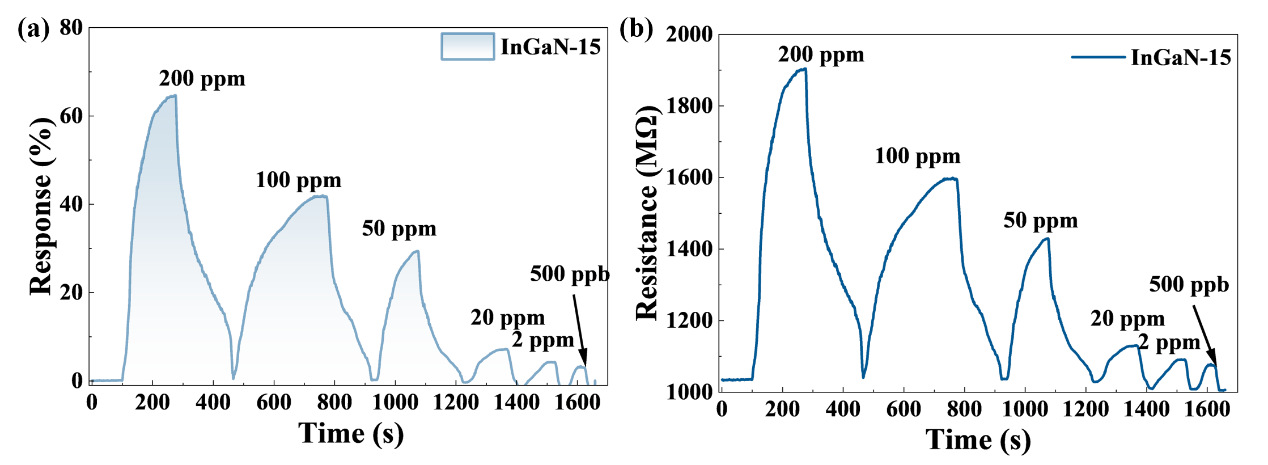


**Supplementary Figure 10**. (a) Response curves of InGaN-15 sensor for NO_2;_ (b) Resistance curves of InGaN-15 sensors forNO_2_.


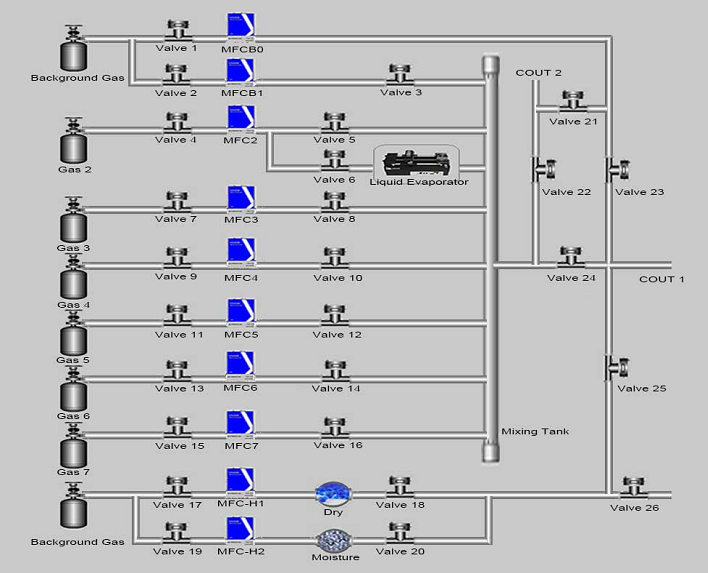


**Supplementary Figure 11.** Dynamic gas distribution path for NO test.

**Supplementary Note 11:** The dynamic gas delivery system developed in this study employs a dual gas source configuration, primarily consisting of high-purity nitrogen (N_2_) and NO standard gas cylinders. The core design of the gas path incorporates two independently controllable lines. The background gas line with high-purity N_2_ is directly regulated through a MFC to provide a stable and adjustable base gas flow. The dilution and mixing line with high-purity N_2_ and NO gases are precisely proportioned using independent mass flow controllers. These gases then undergo initial dilution and thorough mixing within a dedicated mixing chamber.

Ultimately, these two gas streams converge at a downstream confluence point or a main mixing chamber to form the target concentration experimental gas flow output. The entire gas path is connected using PTFE and is equipped with essential pressure reducers, shut-off valves, and exhaust devices to ensure accurate and stable gas composition preparation, as well as safe and controllable operation.

To achieve low-concentration gas testing, gas dilution was performed using a DGL-III Dynamic Gas-Liquid Distribution System. This system features multiple channels capable of dilution ratios ranging from 100 to 5000 times and 10 to 500 times, respectively. This enabled concentration testing of NO_2_ from 200 ppb down to 100 ppt, and NO from 200 ppm down to 2 ppb. For NO testing, gas dilution experiments were conducted using source gases of 10% concentration NO and 10 ppm NO. The flow rates for each dilution channel are detailed in Supplementary Table 3. For low-concentration NO_2_ testing, a 500 ppb using the dilution apparatus. This resulting 500 ppb NO_2_ gas was then employed as the source gas for dynamic dilution testing. The corresponding flow rates for each channel in this dilution process are presented in Supplementary Table 4.

In the gas distribution system, MFCB0 and MFCB1 supplied N_2_, with MFCB0 serving as both the background gas and dilution gas. The target gases were introduced through MFC2 and MFC3. Crucially, MFC2 was capable of achieving dilution factors ranging from 100 to 5000, while MFC3 provided dilution factors from 10 to 500, thus enabling precise control over the concentration of the target analytes.


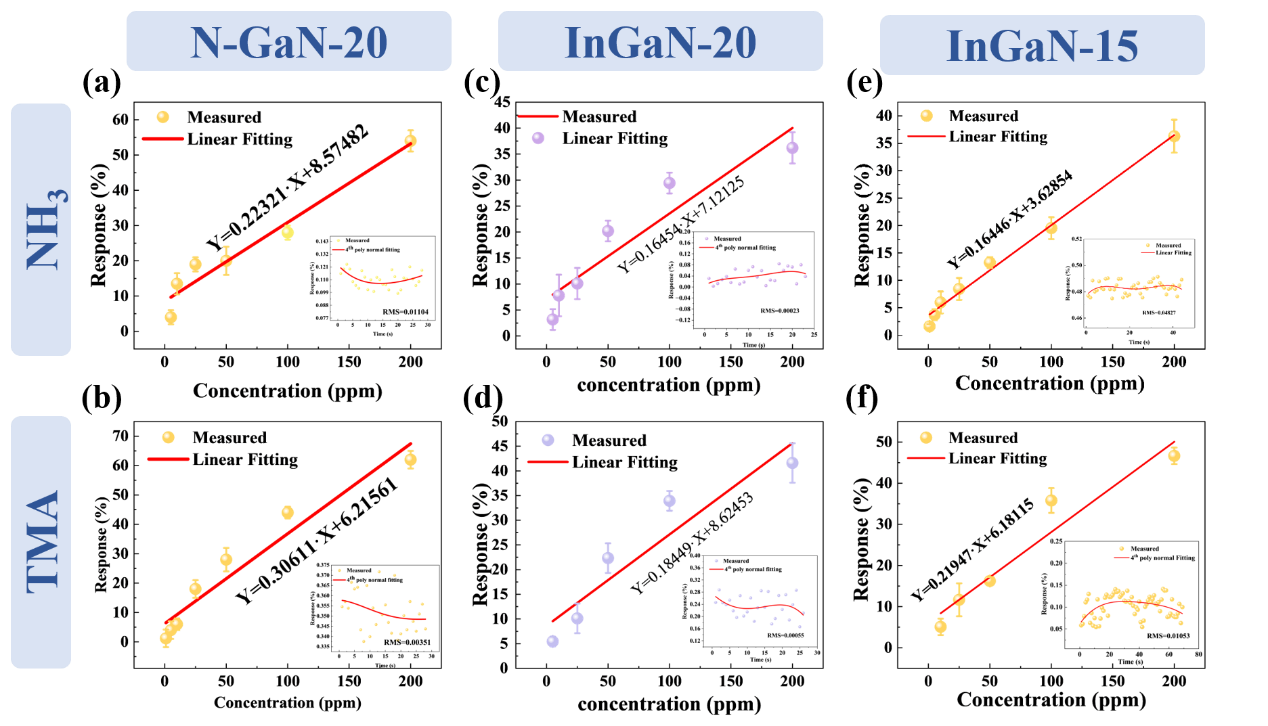


**Supplementary Figure 12.** Linear fit of NH_3_ for (a) InGaN-15, (c) InGaN-20, (e) n-GaN-20 sensors. Linear fit of TMA for (b) InGaN-15, (d) InGaN-20, (f) n-GaN-20 sensors.

**Supplementary Note12:** Due to the challenges associated with detecting the lower limit using liquid ammonia and trimethylamine, a computational approach was considered to estimate the limit of detection (LOD). The fitted curves for the response of InGaN-20, InGaN-15 and n-GaN-20 sensors to NH_3_ and TMA are illustrated in Supplementary Figure 12. In roder to get the lower limit of detection(LOD) of the NH_3_ and TMA. This paper use the equation:

$$\begin{aligned} \mathrm{LOD}\left( \mathrm{ppm} \right)=k\times\frac{\mathrm{RMS}_{\mathrm{noise}}}{\mathrm{slope}} \#\left( 2 \right) \end{aligned}$$

To ensure statistical reliability, all gas concentration tests were conducted with multiple measurements. Triplicate measurements were performed for each concentration level. For the LOD calculation, multiple test results were utilized, and the mean values were subsequently averaged for conducting the fitting analysis. The slope was calculated from the linear section (1~ 200 ppm), the RMS_noise_ is the root-mean-square value of baseline, and k is the expansion factor (a k value of 3 is recommended by IUPAC) ^[4]^. The LOD towards NH_3_ for n-GaN-20 was calculated to be 880.5 ppb where the slope was 0.22321 and RMS_noise_ is 0.01104 (Supplementary Figure 12a). The LOD towards TMA for n-GaN-20 was calculated to be 143.9 ppb where the slope was 0.30611 and RMS_noise_ is 0.00351 (Supplementary Figure 12b). This result indicates the potential capability of the sensor to detect NH_3_ and TMA at a low level of. Based on the same calculation method, the LOD of NH_3_ and TMA for the InGaN-20 sensor was 4.19 ppb (Supplementary Figure 12c) and 8.94 ppb (Supplementary Figure 12d), for the InGaN-15 sensor was 148.3 ppb (Supplementary Figure 12e) and 34.4 ppb (Supplementary Figure 12f).


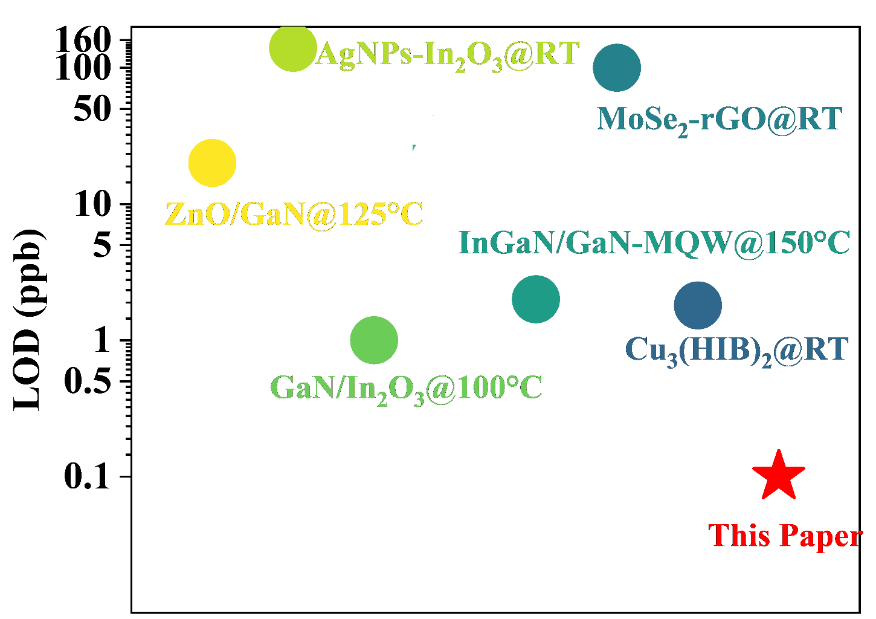


**Supplementary Figure 13.** LOD of NO_2_ for previously reported and this work.


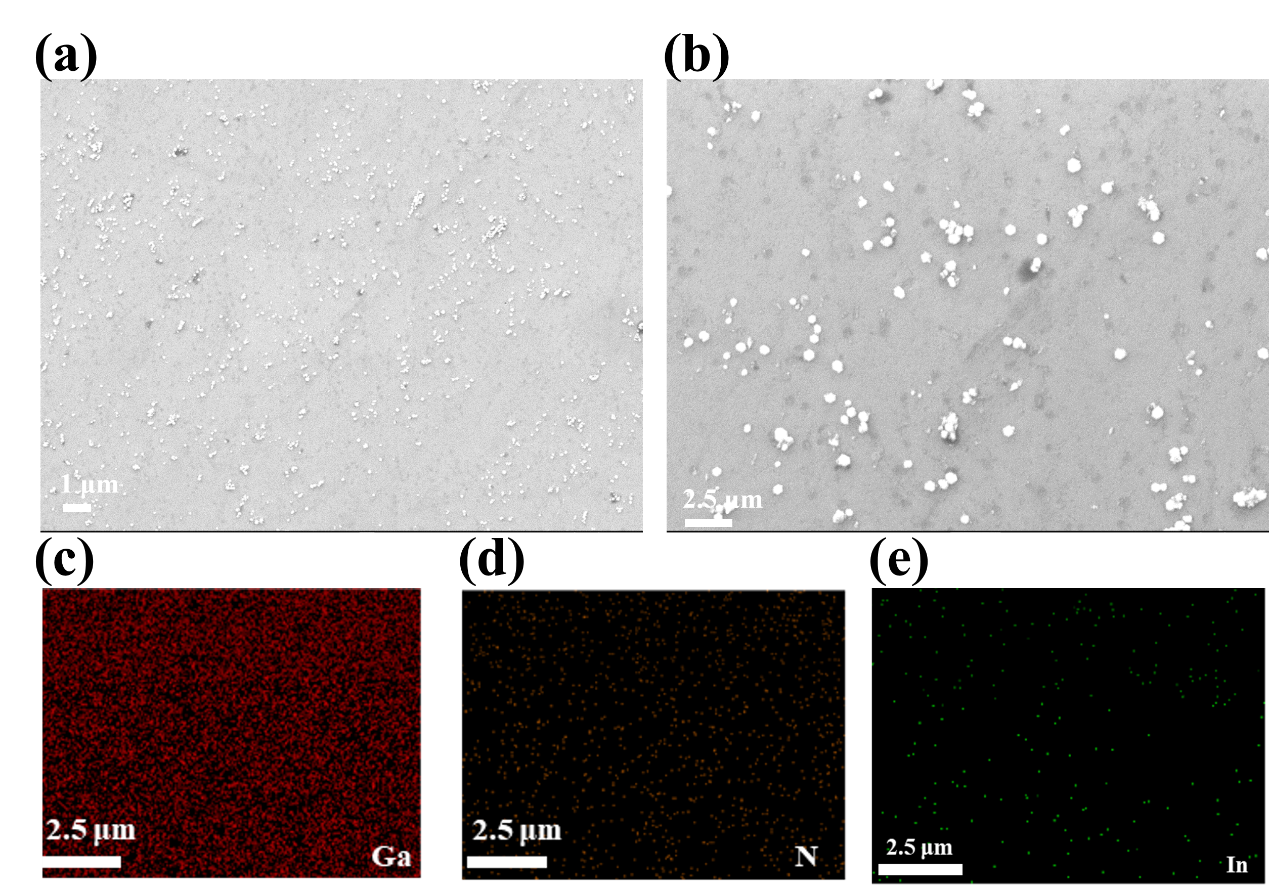


**Supplementary Figure 14.** TEM-EDS of InGaN-High.

**Supplementary Note 14:** The SEM result of InGaN-High is shown as Supplementary Figure 14, the spherical surface structures were observed, with EDS analysis confirming these features is In components. The reason of the spherical structures is because of high In concentration during MOCVD growth will cause the volatilization of In species due to the thermodynamic instability, leading to the formation of In rich droplets through lattice escape and surface segregation.


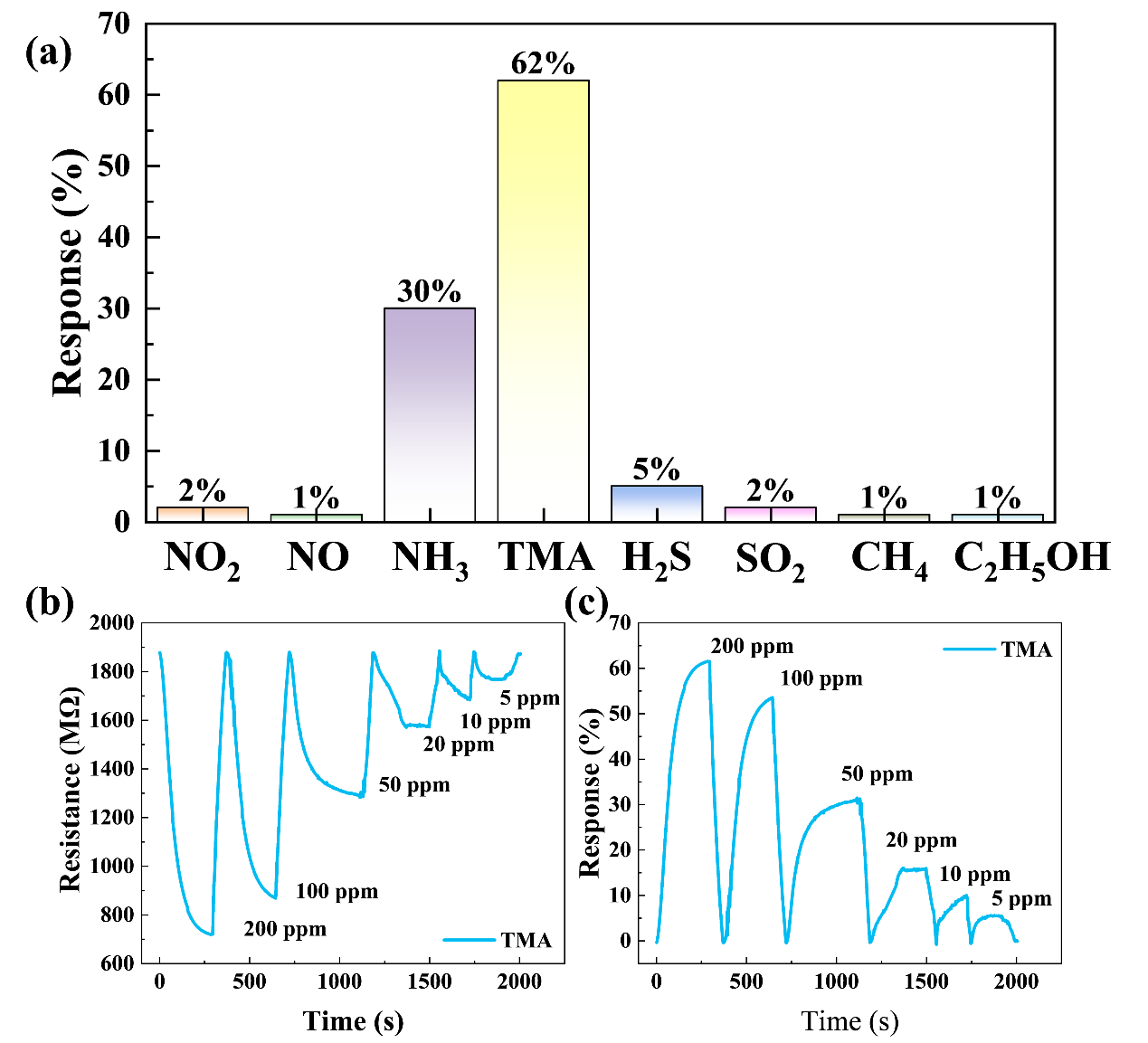


**Supplementary Figure 15.** (a) Selectivity of InGaN-High sensors toward 200 ppm different gases at RT. (b) Resistance curves and (c) Response curves of InGaN-Figh for TMA.

**Supplementary Note 15:** Supplementary Figure 15a shown the selectivity test of the InGaN-High, the InGaN-High exhibited low response to oxidizing gases such as NO_2_, while demonstrate better sensitivity toward reducing species such as TMA, the Supplementary Figure 15b-c showed the response-recovery curve of InGaN-High for TMA. The selectivity test proves the influence of In components, where the incorporation of In components forms an energy level match with the low electron affinity energy of the reducing gas and oxidative gas fail to transfer electronic due to energy level mismatch.


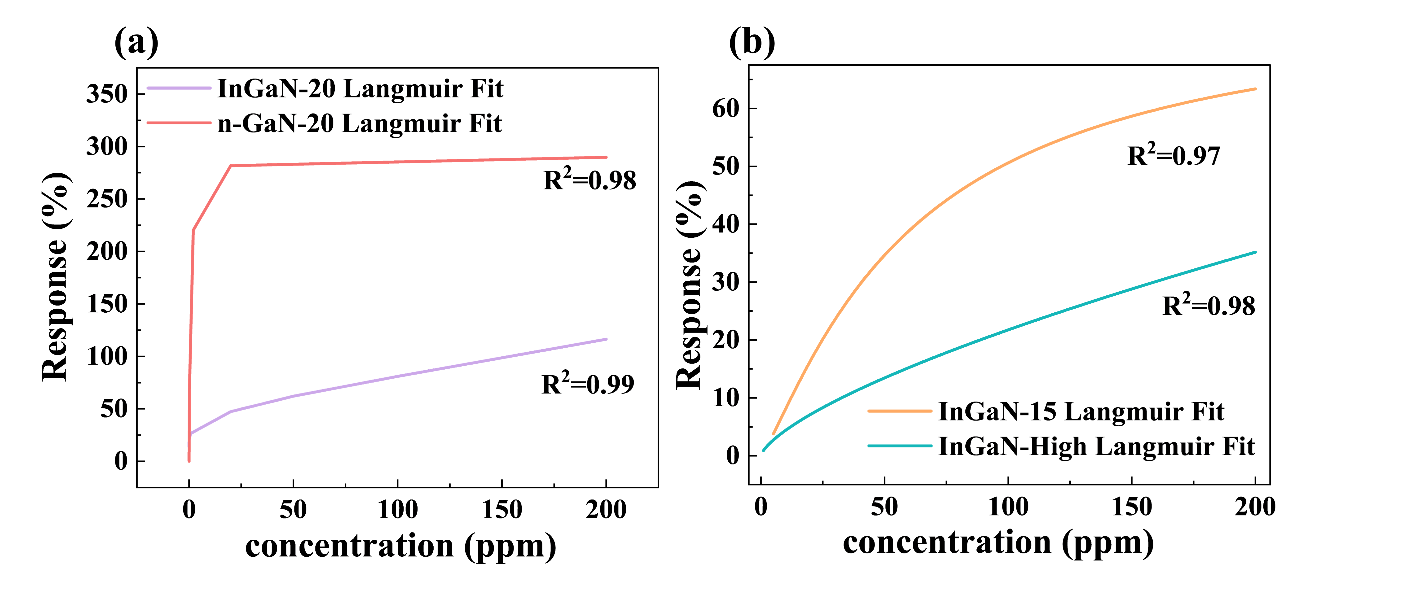


**Supplementary Figure 16.** (a) The Langmuir fit of response and gas concentration of NO_2_ for n-GaN-20 and InGaN-20. (b) The Langmuir fit of response and gas concentration of TMA for InGaN-15 and InGaN-High.

**Supplementary Note 16:** The modified Langmuir coefficients were calculated by using the following equation:

$$\begin{aligned} Q=Q_{m} \times\frac{K\times p^{n}}{1+K\times p^{n}} \#\left( 3 \right) \end{aligned}$$

where Q is the fractional coverage of NO_2_ on the active sites, Q_m_ is the maximum coverage, K is the Langmuir adsorption constant (Pa^-1^), p is the partial pressure of NO_2_, and n is the concentration dependence exponent. The equation can be rewritten as:

$$\begin{aligned} Q=Q_{m} \times\frac{1}{\frac{1}{K\times p^{n}}+1} \#\left( 4 \right) \end{aligned}$$

In this study, the response value (△R/R) and NO_2_/TMA concentration [C (ppm)] were substituted into (4) for simulation purposes. As depicted in Supplementary Figure 16, the result for n-GaN-20 and InGaN-20 demonstrates a strong fit to the Langmuir isotherm model for NO_2_ with R^2^ = 0.88309, 0.9825, respectively. The result for InGaN-15 and InGaN-High demonstrates a strong fit to the Langmuir isotherm model for TMA ags with R^2^ = 0.97319, 0.98809, respectively (Supplementary Figure 16b). The fit results indicate that the observed variation in resistance of the n-GaN-20, InGaN-20, InGaN-15 and InGaN-High can be attributed to the quantity of available active sites.


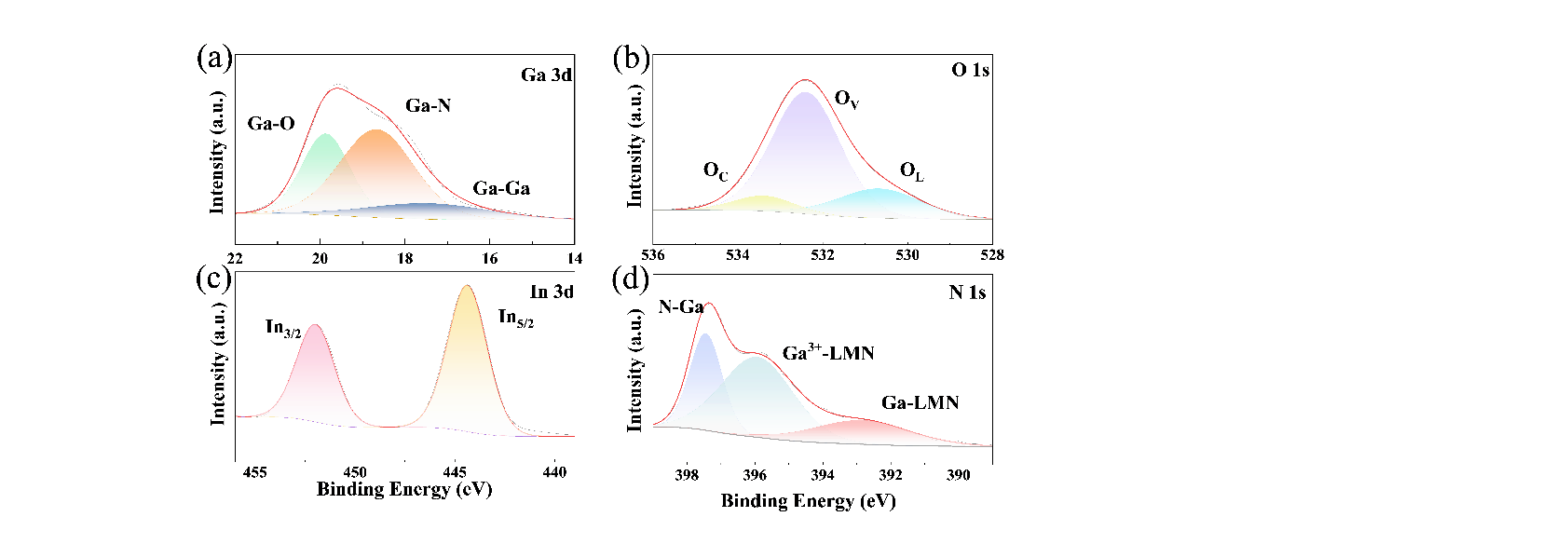


**Supplementary Figure 17** XPS of InGaN-High.

**Supplementary Note 17:** The XPS result of InGaN-High shown on Supplementary Figure 17. Compared to InGaN-20, the InGaN-High has more O_L_ and less O_C_. Because of the incorporation of high concentration of In components reduce the bandgap of GaN and increased electron concentration. In this n-type semiconductor system, surface electron accumulation suppresses oxygen adsorption capability, thereby decreasing chemisorbed oxygen content. Furthermore, In components enhance the formation energy of oxygen vacancies. This will cause a decrease in oxygen vacancies and restrict the generation of surface oxygen adsorption sites. Moreover, forcing more oxygen atoms to incorporate into the lattice by interstitial occupation, causes the increase of the proportion of lattice oxygen.


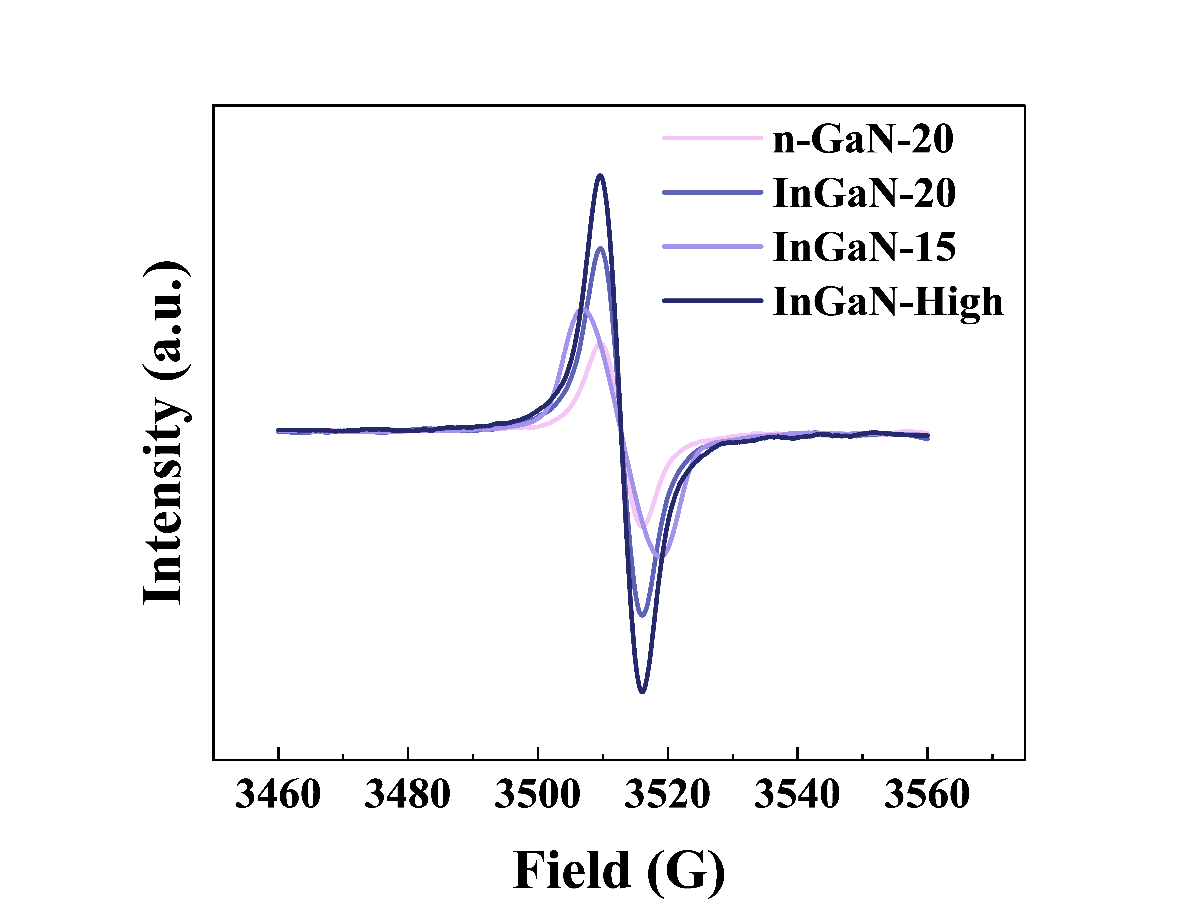


**Supplementary Figure 18.** EPR spectra of vacancy of the sensors built in this work.

**Supplementary Note 18**: In order to reveal the influence of the incorporation of In components for GaN gas sensor, which was studied by EPR measurements. Supplementary Figure 18 displays the EPR spectra of vacancy of the sample of this paper. Notably, all samples exhibit a symmetric derivative peak near the g value of 2.003, with the intensity markedly increasing upon the introduction of In. The incorporation of In components, which possesses a larger atomic radius than Ga causes the lattice expansion and localized compressive stress. To minimize the stress energy of system, the material reshapes structure through stress relief mechanisms, which will generate dislocations, providing nucleation sites for nitrogen vacancies. Meanwhile, the low growth temperature of InGaN suppresses the volatilization of In component. At the same time, the split efficiency of NH_3_ decreases at low temperatures, leading to the lack of active nitrogen, thus increasing the probability of nitrogen vacancy formation. In addition, the incorporation of In components may hinder the ordered arrangement of atoms in the GaN lattice and reduce the atomic mobility, making it difficult for nitrogen atoms to fill the lattice, thus forming stable nitrogen vacancies.


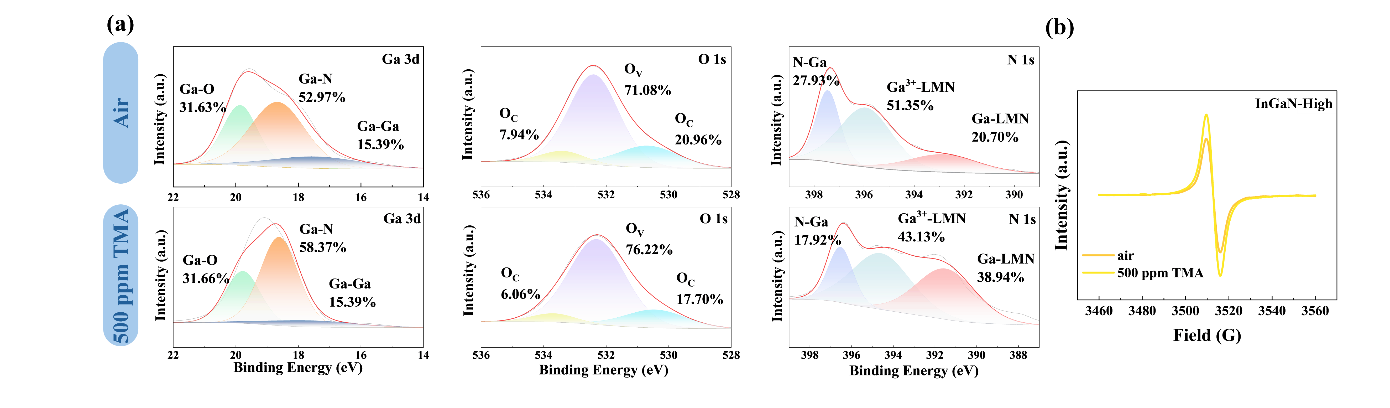


**Supplementary Figure 19.** In-situ characterization of the InGaN-High sensor. (a) In-situ XPS and (b) In-situ EPR monitoring under 500 ppm TMA for 30 min.


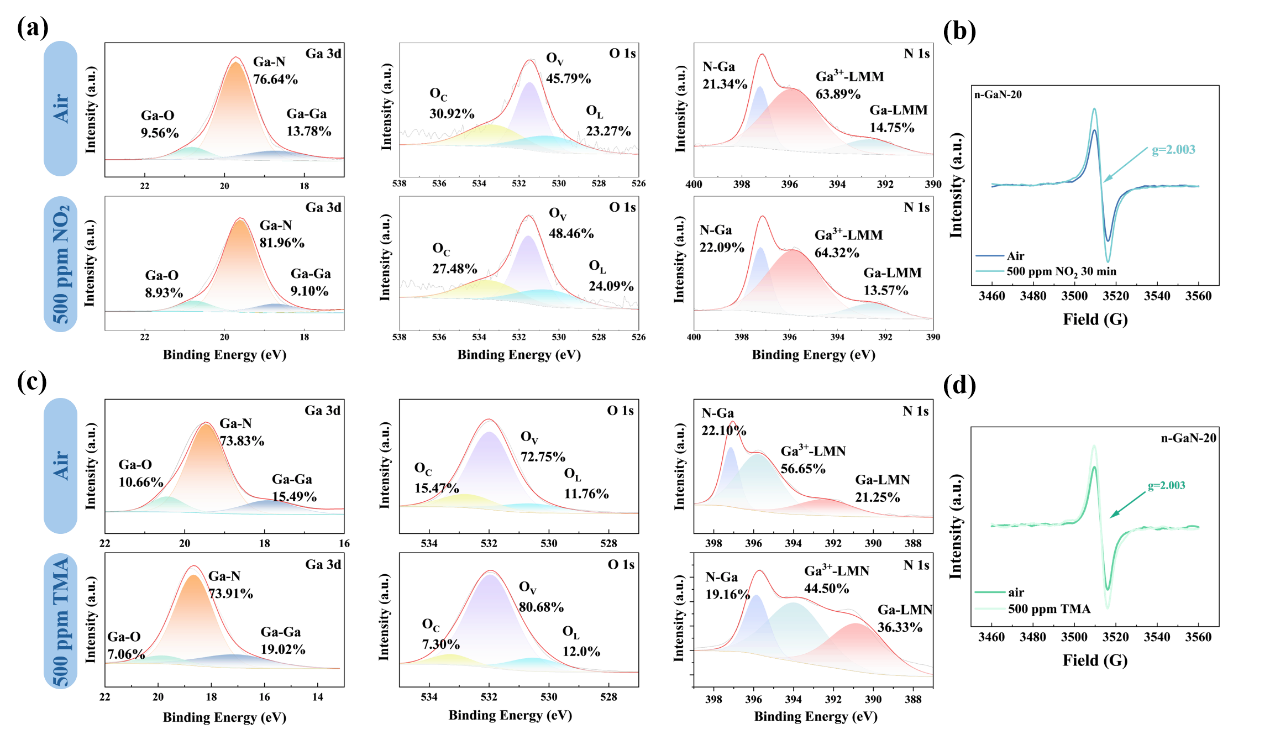


**Supplementary Figure 20.** In-situ characterization of the n-GaN-20 sensor. (a) In-situ XPS and (b) In-situ EPR monitoring under 500 ppm NO_2_ for 30 min. (c) In-situ XPS and (d) In-situ EPR monitoring under 500 ppm TMA for 30 min.


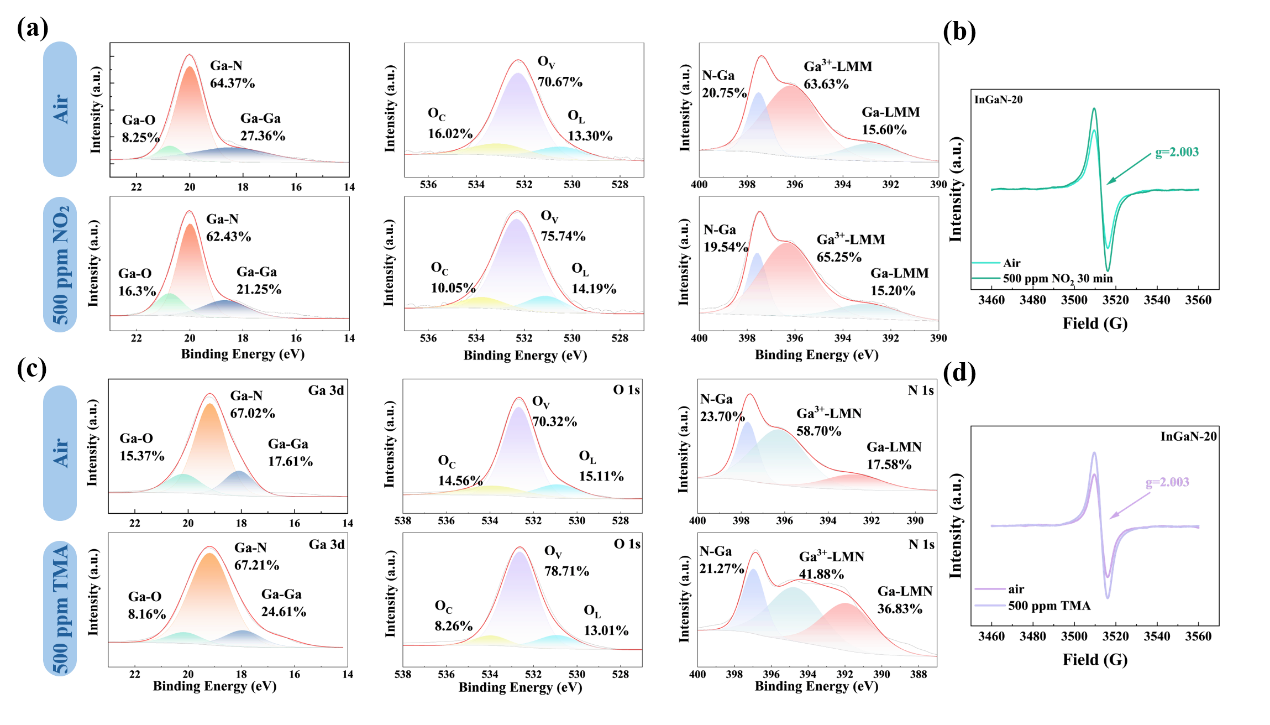


**Supplementary Figure 21.** In-situ characterization of the gas sensing mechanism for InGaN-20 sensors. (a) In-situ XPS and (b) In-situ EPR monitoring under 500 ppm NO_2_ for 30 min. (c) In-situ XPS and (d) In-situ EPR monitoring under 500 ppm TMA for 30 min.


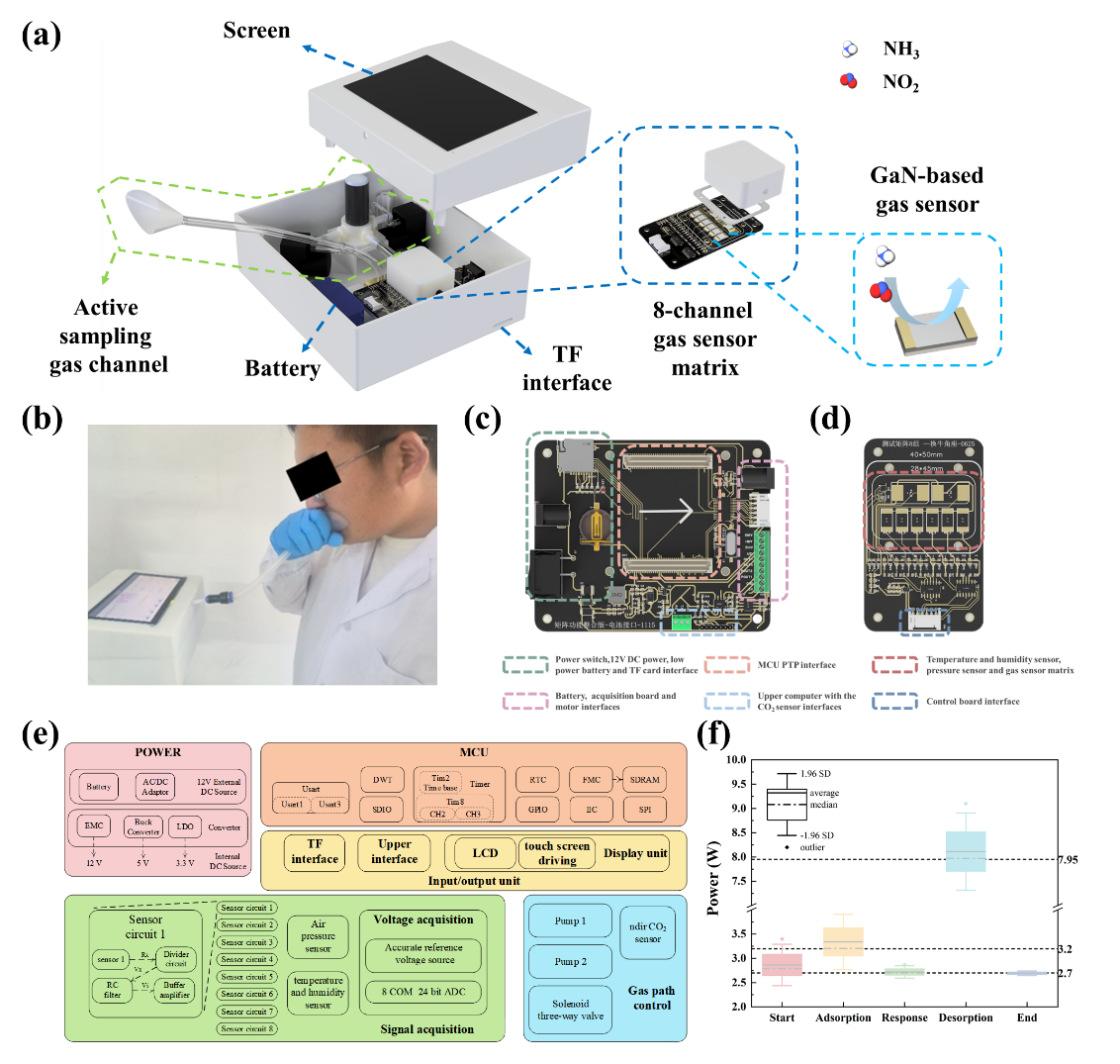


**Supplementary Figure 22.** A smart exhaled air monitoring device for efficient harvesting and continuous exhaled breath. (a) Schematic diagram of explosion of human exhaled breath detection system. (b) Clinical test diagram of exhaled breath system. (c-d) Hardware system PCB board. (e) Hardware circuit diagram of human exhaled breath detection system. (f) Power diagram of human exhaled breath detection system.

**Supplementary Note 22:** The two-channel exhaled breath collection device (TEBCD) including an 8-channel GaN-based sensor matrix circuit and an active sampling gas channel, is shown in Supplementary Figure 22e. The former consisted of a main control unit (MCU, STM32H743IIT6), power unit, input/output unit, signal acquisition unit and gas channel control unit. The MCU is primarily responsible for driving each unit, as well as for data collection and processing. It also has RTC (Real-Time Clock) and WDG (Watchdog) functions, and uses FreeRTOS (10.0.1, FreeRTOS API: CMSIS V1) for task scheduling of each unit. The power supply unit consists of an EMC (Electromagnetic Compatibility) filter, an LDO (Low Dropout Regulator), and a buck converter, and is responsible for providing stable 12V, 5V, and 3.3V power supplies. The input/output unit includes a TF interface, an upper interface, and a screen. The display screen was a channel for man–machine interaction. The MCU drives the screen via LTDC (LCD-TFT Display Controller) and the touch control chip via IIC, using LVGL (Light and Versatile Graphics Library, 8.2.0) to render the UI. Parameters such as pumps, three-way valve and the working state of the system could be set through the screen. At the same time, the screen displayed the waveform of the sensor’s resistance, temperature and humidity and other related signals in real time. The MCU drives the TF card via SDIO, using the FatFs (generic FAT/exFAT filesystem, R0.12c) to store the collected data in XLS format on the TF card.

Targeting the characteristics of semiconductor gas sensors, such as high sensitivity, a wide range of resistance values, and some with high semiconductor resistance (in the order of 100 megohms), the acquisition circuit uses the voltage division method to convert resistance into a voltage signal. After RC filtering, a precision operational amplifier is used to form a voltage follower to amplify the internal resistance of the acquisition circuit. Eight sets of acquisition circuits, together with eight GaN-based gas-sensitive sensors, constitute the sensor matrix. The system converts analog voltage signals into digital signals through an ADC (Analog-to-Digital Converter, ADS1256, 24-bit, 8-channel sensor). The MCU collects the ADC signals at a rate of 10 Hz using the SPI communication protocol. Additionally, due to the susceptibility of semiconductor sensors to environmental interference, the system integrates temperature and humidity sensors, as well as a barometric pressure sensor, within the sensor array to further compensate for the sensor data. In this paper, In order to enhance the detection stability, the sensor array comprises a pair of n-GaN-20, InGaN-20 and InGaN-15.

The device supports power supply via an external power source or a lithium battery, with a battery capacity of 22.2 Wh. Power consumption is shown in Supplementary Figure 22f, with an anomaly at the initial power-on due to high conduction resistance, which leads to higher power consumption. The power consumption for one standard cycle is 0.084 mWh (assuming the exhaled dead space time is 5 seconds). The device can operate continuously for 4 hours and 24 minutes, or 264 cycles. However, during actual testing, due to factors such as changing test personnel and air conditioning, continuous testing was difficult. With a standard preparation time of one minute between tests, the device can operate for 5 hours and 40 minutes, or 170 cycles. This performance meets the requirement for long-term operation in practical scenarios without an external power supply. The designed TEBCD uses a photosensitive resin 3D-printed enclosure, with the structure shown in Supplementary Figure 22a. The overall dimensions of the TEBCD are 204×175×120 mm^3^ (excluding the nozzle part). The unit price of the designed TEBCD is below $300, which is the cost for a single unit; the cost will significantly decrease (by more than 60%) for bulk manufacturing. Compared to most devices with similar functions, this device is smaller in size and more affordable.

The performance of active gas sampling channels hinges on the ability to transfer exhaled gases containing nitrogen-based disease markers into the sensor array. Human beings use the lungs for gas exchange, O_2_, CO_2_, and other gases diffuse and complete the exchange due to differences in partial pressure between the capillaries and the alveolar air. However, during breathing, a portion of the gas remains in the conducting airways and does not participate in gas exchange, which is referred to as the dead space. (The ratio of dead space to tidal volume in normal individuals is about 0.3) ^[5]^. Due to its involvement in gas exchange, alveolar air contains a high concentration of various gas markers in the exhaled breath. By collecting a quantified sample of alveolar air and avoiding the collection of dead space air, it is possible to further eliminate the influence of environmental air and variations in lung capacity among different individuals. The dead space gas and alveolar air in the exhaled breath can be distinguished by the partial pressure of carbon dioxide. Experimental sampling uses an NDIR infrared sensor to detect carbon dioxide concentration, thereby achieving the distinction of alveolar air ^[6]^.

To mitigate noise interference during data acquisition, hardware-level noise suppression was implemented through filtering capacitors in the circuit design, while software-level denoising was achieved by applying the Savitzky-Golay smoothing algorithm to the acquired data.

**
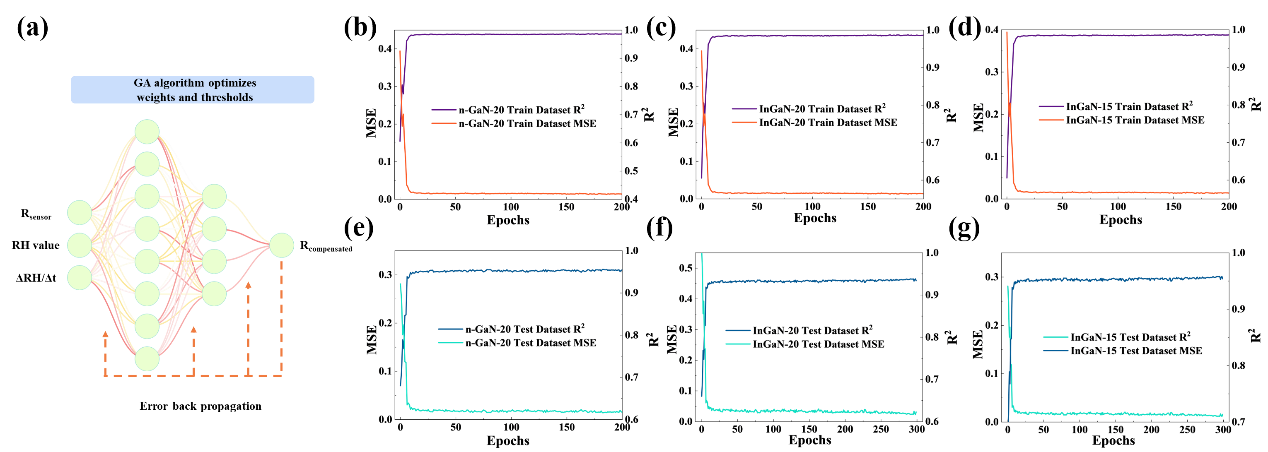
**

**Supplementary Figure 23.** (a) Structure of the humidity compensation model. model train result of MSE and R^2^ result for the (b) n-GaN-20, (c) InGaN-20 and (d) InGaN-15 sensors. model test result of MSE and R^2^ result for the (e) n-GaN-20, (f) InGaN-20 and (g) InGaN-15 sensors

**Supplementary Note 23:** Considering that in exhaled breath detection, the prepared sensors are susceptible to the influence of humidity present in the exhaled gas, a GA-BP neural network is constructed as a sensor humidity compensation model and deployed on an MCU. This model aims to uniformly map the sensor resistance data collected during the acquisition process to the resistance values under the condition of 30% RH in real-time. The training dataset for the model comprises the response data of concentrations from 200 ppm to 1 ppm NO_2_ gas collected from the sensor within a relative humidity range of 30% RH to 90% RH. After the system acquires the resistance signal and the humidity signal, it calculates the rate of humidity change ΔRH/Δt corresponding to the resistance signal. The model utilizes the resistance signal Rsensor, RH value, and the rate of humidity change ΔRH/Δt as the input values for the BP network, with the corresponding resistance value under 30% RH condition R_compensated_ as the model output. The model incorporates two hidden layers and employs Sigmoid as the activation function; a schematic diagram of the model structure is shown in Supplementary Figure 23a. To enhance the convergence efficiency of the model, a Genetic Algorithm is employed for parameter optimization, where the population size is set to 10, chromosomes are encoded in binary form, the crossover probability is set to 0.7, and the mutation probability is set to 0.08. The mean squared error (MSE) progression during training is illustrated in Supplementary Figure 23b-g. The R^2^ exceeds 0.9 across both training and test sets, demonstrating the model’s capability to deliver precise, stable, and reliable humidity response compensation for sensors. The trained GA-BP neural network model requires conversion and optimization through the provided STM32Cube.AI toolchain provided by STMicroelectronics to achieve its embedded deployment on the MCU. During operation, the system collects sensor resistance signals and ambient humidity data in real-time. After preprocessing, these data are input into the onboard neural network model for forward inference, ultimately outputting a humidity-compensated, standardized resistance value. This process effectively mitigates humidity interference, thereby enhancing the accuracy and reliability of gas detection.


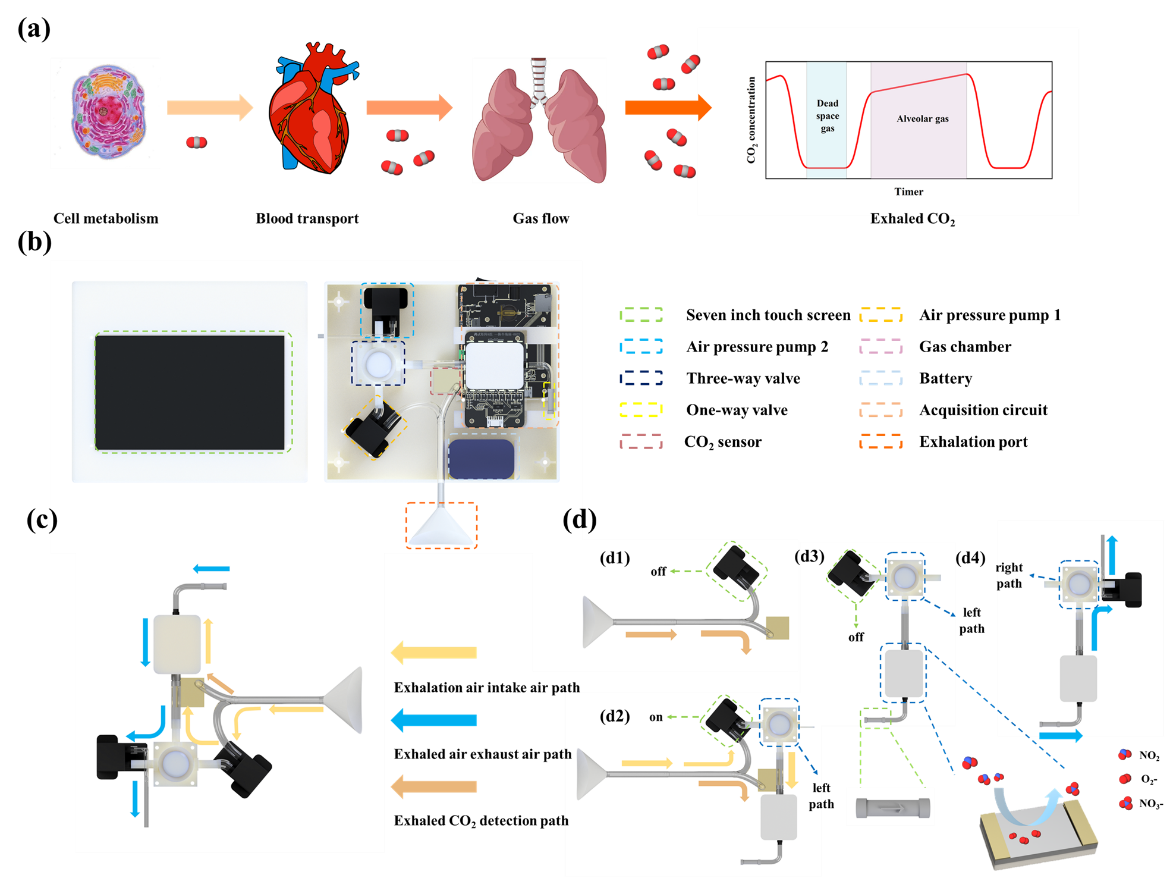


**Supplementary Figure 24.** (a)CO_2_ concentration change diagram. (b) Schematic diagram of explosion of human exhaled breath detection system. (c) exhaled breath system gas path diagram. (d) exhaled breath system gas path acquisition process diagram.

**Supplementary Note 24:** The active gas sampling channel consists of an NDIR CO_2_ sensor, two adjustable-speed air pumps, a three-way valve, a single-way valve, and a gas path, as shown in Supplementary Figure 24. After the test begins, the subject exhales through the mouthpiece. When the CO_2_ concentration exceeds the threshold of alveolar air, the three-way valve is set to the left path, and Pump 1 is turned on to collect exhaled air at a flow rate of 200 ml/min for 10 seconds. Afterward, Pump 1 is turned off. The collected alveolar air reacts with the sensor in the chamber for 20 seconds. Next, the three-way valve is set to the right path, Pump 2 is turned on, and fresh air enters through the one-way valve at a flow rate of 1200 ml/min to clean the chamber. The cleaning lasts for 25 seconds, completing the sampling process. The chamber and gas path are designed using polytetrafluoroethylene (PTFE) with a low friction coefficient and excellent anti-adhesion properties to minimize target gas loss.


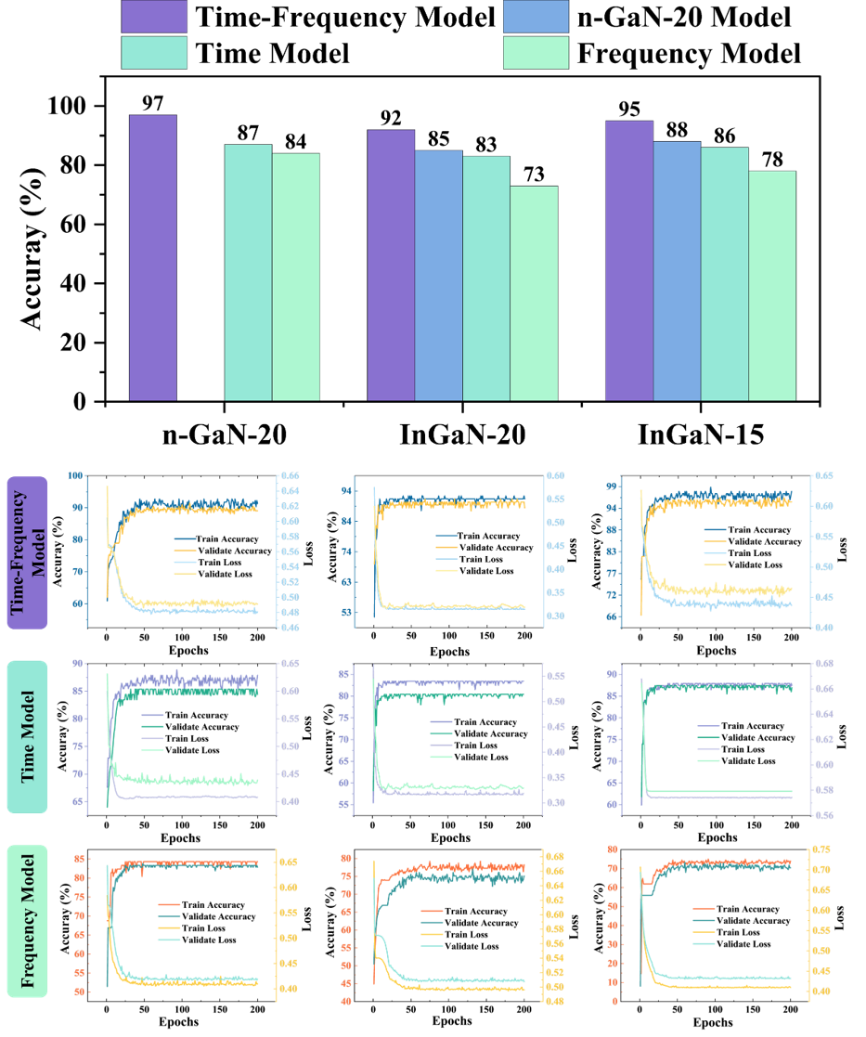


**Supplementary Figure 25.** Model train result and ablation result.


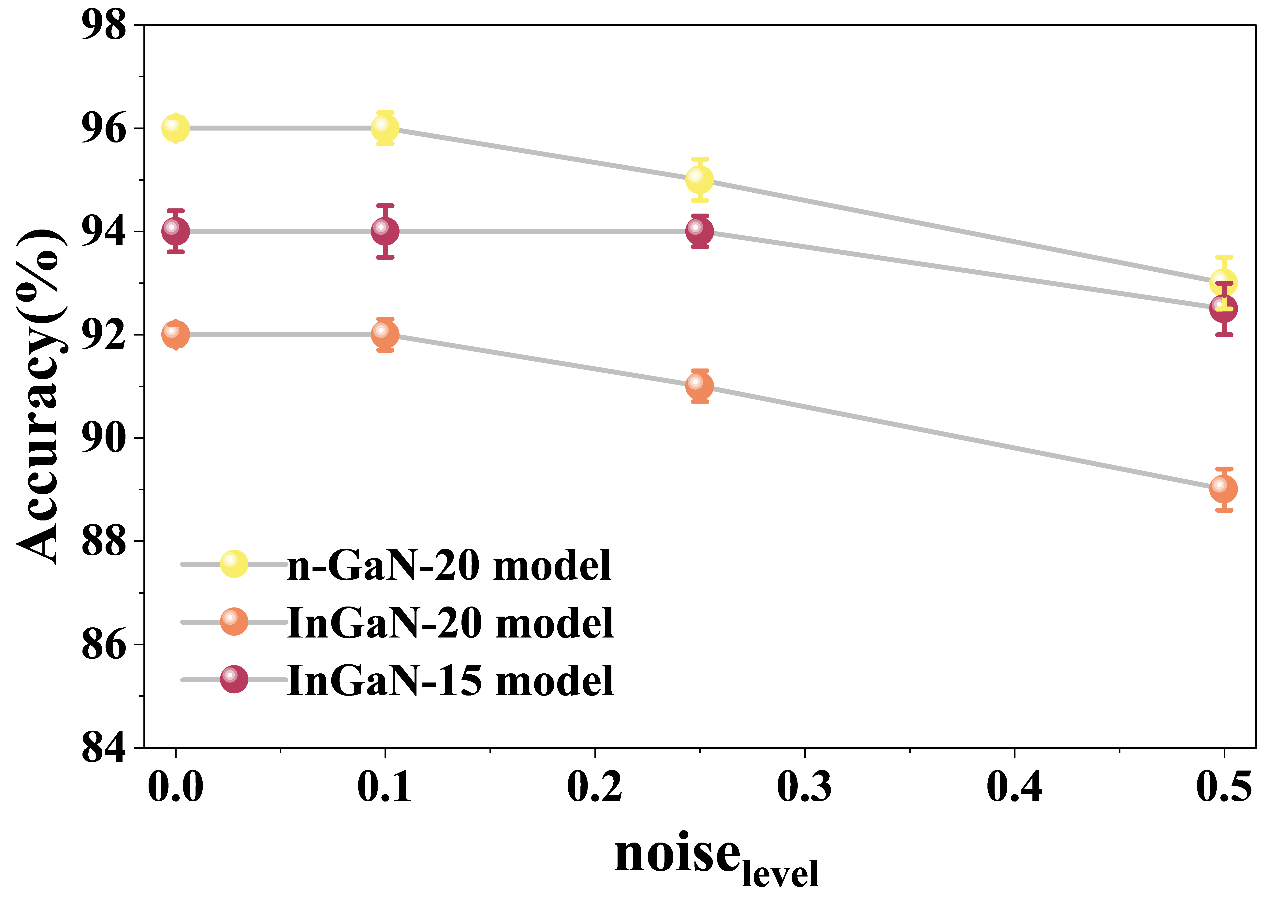


**Supplementary Figure 26** Test results of the model's robustness against Gaussian noise..

**Supplementary Note 26:** To evaluate the model's robustness against interference, this study introduces additive white Gaussian noise of varying intensities to the test data. The noise_level_ is set as a relative intensity coefficient, representing the strength of the added noise relative to the fluctuation of the original data:

$$\begin{aligned} {noise}_{level}={10}^{-\frac{{SNR}_{dB}}{20}} \#\left( 5 \right) \end{aligned}$$

$$\begin{aligned} {SNR}_{dB}=10\log_{10} \left( \frac{P_{signal}}{P_{noise}} \right) \#\left( 6 \right) \end{aligned}$$

P_signal_ is the signal power and P_noise_ is the noise power. The model's performance was tested under noise_levels_ ∈ {0.0, 0.1, 0.25, 0.5}. The test results indicate that as the noise intensity increases, the model's accuracy decreases but remains at a relatively high level. Specifically, at a noise level of 0.5, which corresponds to a SNR_dB_ of 6.02 dB, the accuracy experiences only a 2% reduction. This demonstrates that the model exhibits excellent performance and maintains strong robustness under the interference of moderate-intensity additive white Gaussian noise.

**Supplementary Table 1.** The characterization equipment used in this paper.

| Characterization test | Equipment |
| --- | --- |
| SEM | FESEM; JEOL, JSM-7900F, acceleration voltage: 15 kV |
| TEM | Thermo Scientific Talos F200X |
| XPS | Thermo Scientific, ESCALAB Xi+ using Al- Kα as the source |
| XRD | Malvern Panalytical Aeris with Cu-Kα radiation (λ = 1.54056 Å) |
| Raman | Renishaw Invia with an excitation wavelength of 532 nm. |
| EPR | BRUKERA300 |
| In-silt XPS | Thermofisher escalab 250xi |
| AFM | Dimenson ICON |
| PL | Edinburgh FLS-1000 |

**Supplementary Table 2.** Carrier mobility for the n-GaN-20, InGaN-20 and InGaN-20.

|  | Carrier type | carrier mobility (cm^2^/Vs) |
| --- | --- | --- |
| n-GaN-20 | n | 169.43276 |
| InGaN-20 | n | 13.93729 |
| InGaN-15 | n | 3.09173 |

**Supplementary Table 3.** The flow rates for each dilution channel of NO dynamic gas-blending system.

|  | Source | MFCB0 | MFCB1 | MFC2 | MFC3 | Total |
| --- | --- | --- | --- | --- | --- | --- |
| 200 ppm | 10% NO | 500 | 1 | 499 | \ | 1000 |
| 100 ppm | 10% NO | \ | 1 | 999 | \ | 1000 |
| 50 ppm | 10% NO | \ | 0.5 | 999.5 | \ | 1000 |
| 25 ppm | 10% NO | \ | 0.25 | \ | 999.75 | 1000 |
| 2 ppm | 200 ppm NO | 900 | 1 | \ | 99 | 1000 |
| 500 ppb | 200 ppm NO | 600 | 1 | \ | 399 | 1000 |
| 250 ppb | 10 ppm NO | 990 | 0.25 | \ | 9.75 | 1000 |
| 50 ppb | 10 ppm NO | 800 | 1 | 199 | \ | 1000 |
| 25 ppb | 10 ppm NO | 800 | 0.5 | 199.5 | \ | 1000 |
| 10 ppb | 10 ppm NO | 0 | 1 | 999 | \ | 1000 |
| 2 ppb | 10 ppm NO | 0 | 0.2 | 999.8 | \ | 1000 |

**Supplementary Table 4.** The flow rates for each dilution channel of NO_2_ dynamic gas-blending system.

|  | Scource | MFCB0 | MFCB1 | MFC2 | MFC3 | Total |
| --- | --- | --- | --- | --- | --- | --- |
| 200 ppb | 500 ppb NO_2_ | 500 | 200 | 300 | \ | 1000 |
| 100 ppb | 500 ppb NO_2_ | 500 | 100 | 400 | \ | 1000 |
| 50 ppb | 500 ppb NO_2_ | 900 | 10 | 90 | \ | 1000 |
| 20 ppb | 500 ppb NO_2_ | 900 | 4 | 96 | \ | 1000 |
| 2 ppb | 500 ppb NO_2_ | 900 | 2 | 98 | \ | 1000 |
| 200 ppt | 500 ppb NO_2_ | 20 | 2 | 978 | \ | 1000 |
| 100 ppt | 500 ppb NO_2_ | 20 | 1 | 979 | \ | 1000 |

**Supplementary Table 5.** Accuracy, recall and F1 for each sensor of three models. (C1: n-GaN-20 sensor, C2: InGaN-20 sensor, C3: InGaN-15 sensor,)

|  | Train Dataset | | | | | | | | |
| --- | --- | --- | --- | --- | --- | --- | --- | --- | --- |
|  | Accuracy | | | Recall | | | F1 | | |
|  | C1 | C2 | C3 | C1 | C2 | C3 | C1 | C2 | C3 |
| TFAM | 92 | 92 | 95 | 0.1 | 0.13 | 0.13 | 0.87 | 0.85 | 0.86 |
| TAM | 87 | 83 | 86 | 0.15 | 0.14 | 0.14 | 0.85 | 0.84 | 0.85 |
| FAM | 84 | 77 | 73 | 0.16 | 0.25 | 0.21 | 0.82 | 0.81 | 0.83 |

**Supplementary Table 6.** Accuracy, recall and F1 for each sensor of three models. (C1: n-GaN-20 sensor, C2: InGaN-20 sensor, C3: InGaN-15 sensor,)

|  | Validation Dataset | | | | | | | | |
| --- | --- | --- | --- | --- | --- | --- | --- | --- | --- |
|  | Accuracy | | | Recall | | | F1 | | |
|  | C1 | C2 | C3 | C1 | C2 | C3 | C1 | C2 | C3 |
| TFAM | 89 | 89 | 94 | 0.15 | 0.18 | 0.16 | 0.85 | 0.82 | 0.83 |
| TAM | 8 5 | 80 | 85 | 0.2 | 0.21 | 0.18 | 0.81 | 0.79 | 0.84 |
| FAM | 82 | 74 | 70 | 0.19 | 0.31 | 0.26 | 0.82 | 0.78 | 0.79 |

**Supplementary Reference**

[1] D. Li, D. Han, Y. Chen, Z. Liu, X. Liu, L. Liu, X. Han, X. He, S. Sang, *Sensors Actuators B: Chemical* **2022**, *371*, 132583.

[2] X. Wu, S. Shi, J. Jiang, D. Lin, J. Song, Z. Wang, W. Huang, *Advanced Materials* **2025**, *37* (13), 2419159.

[3] S. Nongthombam, N. A. Devi, S. Sinha, R. Bhujel, S. Rai, W. Ishwarchand, S. Laha, B. P. Swain, *Journal of Physics Chemistry of Solids* **2020**, *141*, 109406.

[4] A. M. Committee, *Analyst* **1987**, *112* (2), 199.

[5] J. Petersson, R. W. Glenny, *European Respiratory Journal* **2014**, *44* (4), 1023.

[6] T. A. Vincent, J. Gardner, *Sensors Actuators B: Chemical* **2016**, *236*, 954.
